# Pandemic-induced increase in adjustment disorders among postpartum women in Germany

**DOI:** 10.1186/s12905-023-02638-z

**Published:** 2023-09-12

**Authors:** K. Tsoneva, N. Chechko, E. Losse, S. Nehls, U. Habel, A. Shymanskaya

**Affiliations:** 1 Department of Psychiatry, Psychotherapy and Psychosomatics, Faculty of Medicine, RWTH Aachen University, Pauwelsstraße 30, 52074 Aachen, Germany; 2 Institute of Neuroscience and Medicine, JARA-BRAIN Institute Brain Structure and Function, Jülich Research Centre, INM-10 Jülich, Germany; 3 Institute of Neuroscience and Medicine, Brain & Behavior (INM-7), Research Center Jülich, Jülich, Germany

**Keywords:** Postpartum adjustment disorder, Postpartum depression, Postpartum period, COVID-19

## Abstract

**Background:**

The current paper analyzed the effect of the pandemic-induced lockdown on maternal mental health during the first 12 postpartum weeks in Germany.

**Methods:**

In this cohort study, we compared the participants’ anamnestic backgrounds and the results of psychological tests, measuring stress levels, depressive symptoms and attachment. The 327 participants were divided into two groups with one representing the “pre-COVID” sample and the other the “lockdown” sample. We performed multiple comparisons, investigating the distribution of diagnoses and the correlating risk profiles between the two cohorts.

**Results:**

Our analysis showed a significant difference between the two cohorts, with a 13.2% increase in the prevalence of adjustment disorders (AD), but not postpartum depression (PPD), in the first 12 weeks postpartum. However, during the pandemic, women with AD had fewer risk factors compared to their pre-pandemic counterparts. In the “lockdown” cohort, a tendency toward higher stress and lower mother-child attachment was observed in AD.

**Conclusions:**

In sum, we observed some negative impact of the pandemic on maternal mental health. The lockdown might have contributed to an increase in the number of cases involving AD in the postpartum period. The prevalence of PPD (ca. 6–10%), on the other hand, was not affected by the lockdown. Thus, the effect of COVID-19 on maternal mental health might not, after all, have been as severe as assumed at the beginning of the pandemic.

**Supplementary Information:**

The online version contains supplementary material available at 10.1186/s12905-023-02638-z.

## Background

With the outbreak of the COVID-19 pandemic in 2020, many public health experts voiced concern over a possible mental health crisis of great magnitude [1, 2]. Studies from across the world suggested increased odds for psychiatric morbidity during the pandemic [3–7], especially given that the pandemic amplified some risk factors such as financial insecurity [8, 9], unemployment [10], and social isolation [11, 12]. Additionally, the sanitary crisis played a crucial role as a unique stressor [13].

On 22nd March 2020, Germany imposed a partial lockdown and extensive protective measures against the spread of COVID-19 [14]. A rise in stress-induced psychopathology was expected due to the widespread societal changes as a result of the severe lockdown measures [15]. In Germany, public health authorities had been generating a continuous flow of data on the prevalence of mental health disorders prior to the COVID-19 pandemic [16–19]. German studies analyzing the COVID-19-related effect on mental disorders reported a significant decrease in psychopathologies in the initial phase of the pandemic [20–22]. However, [23] reported a slight increase in the depressive symptoms, describing financial distress as a determining factor.

While these results might have been representative of the general population, the circumstances involving the vulnerable population subgroups needed to be further explored [21]. Depression was reported to be twice as prevalent among reproductive-age women as men [24–28]. Given the elevated risk of psychiatric pathologies during the postpartum period [29–31], both instrumental assistance and emotional support from partner and family are critically important [32]. The severe lockdown-induced restrictions [14], which curtailed social contacts across the board, might have had a particularly negative effect on the everyday life of young mothers. Studies examining the pandemic’s effect on maternal mental health indicated a significant increase in the frequency of physician visits due to perinatal mental disorders [33] as well as a rise in postpartum depression in multiple countries [34, 35]. More recent longitudinal studies and meta-analyses, on the other hand, have drawn a picture of relative resilience with respect to mental health during the pandemic [36]. The findings of Shevlin et al. [37] suggest that, among UK adults, the prevalence of anxiety and depression remained stable across the first 4 months of the pandemic, while COVID-19-related PTSD fell between April and July 2020. The reported prevalence of anxiety and depression was not markedly higher than in previous epidemiological surveys [38]. While there were indications of a temporary effect of pandemic-related stress on mental health, there is no evidence of any long-term effect, with most affected individuals having rapidly adapted to the situation. For instance, women who initially reacted with increased levels of anxiety and depression, quickly adjusted themselves to the circumstances and were among those showing the fastest mental health improvements in the first few weeks of the lockdown [39].

Adjustment disorder (AD) and postpartum depression (PPD) are the most frequent affective conditions seen in the postpartum period. Occurring in 5 to 15% of cases, PPD represents a depressive disorder with the onset around childbirth or within the first four weeks postpartum [40, 41]. AD, which is a stress-related, subclinical affective condition [40], affects up to 12% of new mothers [42] and is largely self-remitting [3, 42]. Both conditions are related to the hormonal changes during pregnancy and the postpartum period [42] occurring more frequently in women with a history of mental illness, psychological and social distress, and experience of stressful life events [42, 43].

Exploring the possibility of a link between sociodemographic and COVID-19-related factors as well as the effects of the pandemic-related restrictive measures on depression and anxiety symptoms, a German study [44] found an association between diminished social contacts and poorer mental health in the overall population. To our knowledge, however, there has been no research addressing the effect of lockdown and pandemic-related restriction on mental health of German mothers in the first few postpartum weeks. Similar studies in the USA, Turkey and China recruited heterogeneous groups of new mothers at differing time points (i.e. six months to one year after childbirth), collecting all relevant data online [34, 35, 45–48]. Also, the diagnoses in these studies were based only on online self-assessment questionnaires such as the Edinburgh Postnatal Depression Scale (EPDS) [49], which, used broadly, likely classifies subclinical conditions as pathological and cannot, therefore, accurately distinguish AD from PPD [50]. Thus, the use of self-reports alone would lead to an overestimation of the prevalence of depression. Moreover, often the EPDS was assessed only at one time point throughout the postpartum period, ignoring the dynamics of postpartum mental illness and possibly convolving AD with PPD [35, 45–48].

The purpose of our study was to investigate the influence of the pandemic-induced lockdown on the prevalence, occurrence and short-term temporal development of AD and PPD in new mothers in Germany. To that end, we observed the depressed mood, the quality of mother-child attachment and stress levels in a cohort of 327 new mothers for the first 12 weeks postpartum. Around two-thirds of these women completed their participation prior to the nationwide lockdown, forming the “pre-COVID” cohort in our study, while the rest of the participants were included in the so-called “lockdown” cohort. A number of biological and psychosocial/environmental factors that may contribute to the development of depression (e.g., baby blues, stressful life events (SLE), history of mental illness, premenstrual syndrome (PMS)) were assessed at enrolment.

We aimed at estimating the prevalence of AD and PPD among those who gave birth during the pandemic-related restrictions and compared it with that of the “pre-COVID” cohort. In non-depressed (ND), AD and PPD groups, we also sought to compare the risk profiles, depressed mood scores, mother-to-child attachment, and stress levels in (i) the whole sample independent of the lockdown, and (ii) based on the lockdown status, comparing “lockdown” vs. “pre-COVID” cohorts. Based on the available literature, we hypothesized that there would be a significant increase in the prevalence of depressive disorders (both AD and PPD) in the “lockdown” sample. Independent of the lockdown status, we expected both PPD and AD to be associated with a number of risk factors [42]. We hypothesized, also, that women in the “lockdown” cohort would have higher stress levels [51] and would more frequently develop AD or PPD without typical PPD or AD risk profiles, confirming the substantial mental distress induced by the pandemic [52]. Thus, even in the absence of the increased risk to develop an affective postpartum disorder, the situation of the lockdown itself would put women at a higher risk of developing either AD or PPD.

## Materials and methods

### Participants

A cohort of 327 women was recruited between November 2018 and December 2020 within the first 6 days of childbirth at the obstetrics ward of the University Hospital of Aachen as part of the ongoing longitudinal study related to early detection of postpartum depression (RiPoD: Risk of postpartum Depression) [53].

To investigate the influence of the pandemic on mental health of women after childbirth, we extracted two samples – one prior to and one during the pandemic. Out of the 327 women, 211 were interviewed prior to the lockdown (interviewed before 22.03.2020) and were placed in the “pre-COVID” cohort. The remaining 116 women were recruited following the imposition of the nationwide lockdown. Interviewed after 22.03.2020, they constituted the “lockdown” cohort.

Sufficient knowledge of the German language was a prior requirement for participation in this study, as all the questionnaires and clinical interviews were in German. The exclusion criteria included age outside the 18–45 range, current depression, abuse of alcohol, drugs, psychotropic substances, antidepressant or antipsychotic medication during pregnancy, history of psychosis or manic episodes. Women who suffered serious complications at childbirth, such as preeclampsia, eclampsia and HELLP-syndrome, were also not included in the study. The infant-related exclusion criteria were genetic defects (e.g. trisomies), premature birth (< 29 + 0 week) and low birth weight (< 1500 g). Written informed consent was acquired from each participant prior to recruitment. The study was performed in accordance with the declaration of Helsinki and approved by the Institutional Review Board of the Medical Faculty, RWTH Aachen University (reference number EK208-15). While the recruitment of new participants was paused between 08.03.2020 and 12.05.2020 due to the pandemic-related contact restrictions, the clinical interviews had continued. Apart from adapting the study to the government’s hygiene measures, such as the wearing of medical masks and maintaining a minimum of 1.5 m distance, no changes were made to the study design during the lockdown, mitigating any potential bias during data collection.

### Procedure and questionnaires

**Fig. 1:**
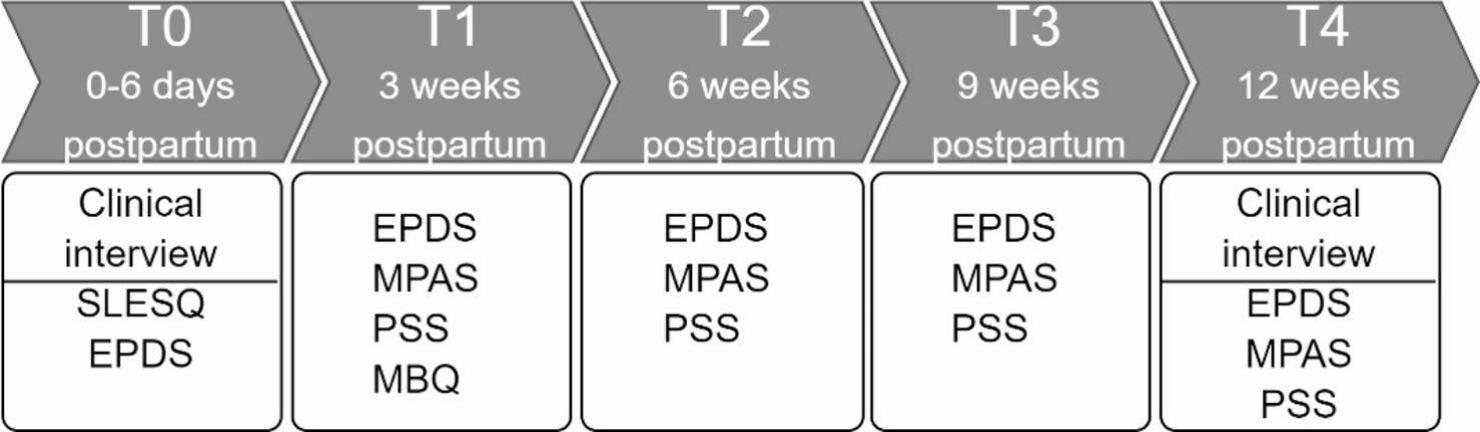
Temporal design of the RiPoD study procedure. The assessments were performed 3 weeks apart, with T0 being immediately after childbirth Note: SLESQ: Stressful Live Events Screening Questionnaire; EPDS: Edinburgh Postpartum Depression Scale; MPAS: Maternal Postnatal Attachment Scale; PSS: Perceived stress scale; MBQ: Maternity Blues Questionnaire

The study design was based on a 12-week postpartum follow-up, with the data being collected at five time points three weeks apart (T0 - T4), beginning a few days after childbirth. At T0, a clinician performed a clinical interview along with a screening for depressive symptoms using the Edinburgh Postnatal Depression Scale (EPDS) questionnaire [49]. A clinical-anamnestic screening was also conducted at this point, which helped obtain information on socio-demographic status, pregnancy, and family history of mental illness. At the time of enrolment, the participants filled out the Stressful Live Events Screening Questionnaire (SLESQ) to help assess encounters with SLE [54]. A further survey was completed to determine if the participants qualified for the Premenstrual syndrome (PMS) [55]. The mother-to-child attachment was measured using the Maternal Postnatal Attachment Scale (MPAS) [56], a self-report instrument on which elevated values indicate better attachment. Perceived psychological distress was measured using Cohen’s Perceived Stress Scale (PSS), with a 10-item self-report measure [57]. At three weeks postpartum, the mothers were given an additional Maternity Blues Questionnaire (MBQ) to help detect the psychological symptoms experienced in the first week after delivery [58]. At 3 weeks (T1), 6 weeks (T2), and 9 weeks (T3) postpartum, the participants were asked to log in to the online survey platform “Survey Monkey” to enable the assessment of depressive symptoms (EPDS), mother-to-child attachment (MPAS) and stress (PSS) for the preceding three weeks. At 12 weeks postpartum (T4), an assessment of EPDS, MPAS and PSS was performed and a clinical interview was conducted by a trained psychologist or psychiatrist. The participants were assigned to the ND, PPD and AD groups based on the DSM-5 criteria. The study procedure is shown in Fig. 1.

### Missing data

Before investigating the distribution of diagnoses in the two cohorts and their risk factor profiles, we investigated the distribution of missing data across the whole population. Since no patterns were detected in the missing data, we assumed that the data were missing at random. The distribution of missing data is provided in the Supplementary Material (SM Fig. 1). The data were imputed using the mice package [59–61] (RStudio Version 1.4.1106 for macOS) with the following imputation algorithms selected based on the data type: for numerical data – pmm, for ordered – polr, for binary – logreg, for factor – polyreg.

### Differences in prevalence of ND, AD and PPD between the “pre-COVID” and “lockdown” cohorts

The study participants were divided into three groups based on the diagnosis they obtained after 12 weeks: ND (non-depressed participants), women who remained healthy; AD, women who developed adjustment disorder; and PPD, women who developed postpartum depression. The PPD and AD prevalence in both the “pre-COVID” and “lockdown” cohorts was calculated and subsequently compared. The prevalence in both cases was estimated using 95% confidence intervals generated with the bootstrap method with 10^5^ replicates from SPSS [62] (IBM SPSS Statistics, Version 26 for Windows).

### Differences in anamnesis within and between cohorts

A comparison of the anamneses between the “pre-COVID” and “lockdown” cohorts without a diagnostic separation is presented in Table 1. Table 2 contains a detailed description of the study population without differentiation between the “pre-COVID” and “lockdown” cohorts and differences in clinical-anamnestic and demographic data between the diagnostic groups. Additionally, to average values across the diagnoses, the results of the chi-squared or Fishers exact tests are provided to facilitate diagnosis comparisons for possible differences. The calculations were performed using a package finalfit [63] (RStudio Version 1.4.1106 for macOS) and MS Excel 2017 [64] (Microsoft Excel for Mac, Version 15.34). p-values less than 0.001 are considered statistically significant. Full anamneses of all three diagnostic subgroups in the “pre-COVID” and “lockdown” cohorts are provided in Tables SM2 and SM3. A further comparison was performed to investigate if the groups differed in anamnesis between “pre-COVID” and “lockdown” cohorts (SM4 columns “pre-COVID”-cohort and “lockdown”-cohort). Also, we investigated differences within the diagnoses between the “pre-COVID” and “lockdown” cohorts (SM5, SM6 and SM7), performing a comparison without diagnostic separation between the cohorts (see SM8).

### Differences in depressed mood, mother-to-child attachment, stress scores and temporal trajectories within and between cohorts

Lastly, we investigated the magnitude and temporal development of the depressed mood, mother-to-child attachment, and stress scores in (1) the whole cohort for the three diagnostic subgroups and (2) the “pre-COVID” and “lockdown” cohorts for the three diagnostic subgroups. We also qualitatively compared the temporal development of trajectories of the mother-to-child attachment, depressed mood, and stress scores, which were visualized using Microsoft Excel for Windows OS.

## Results

### Differences in prevalence of ND, AD and PPD between the “pre-COVID” and “lockdown” cohorts

**Fig. 2:**
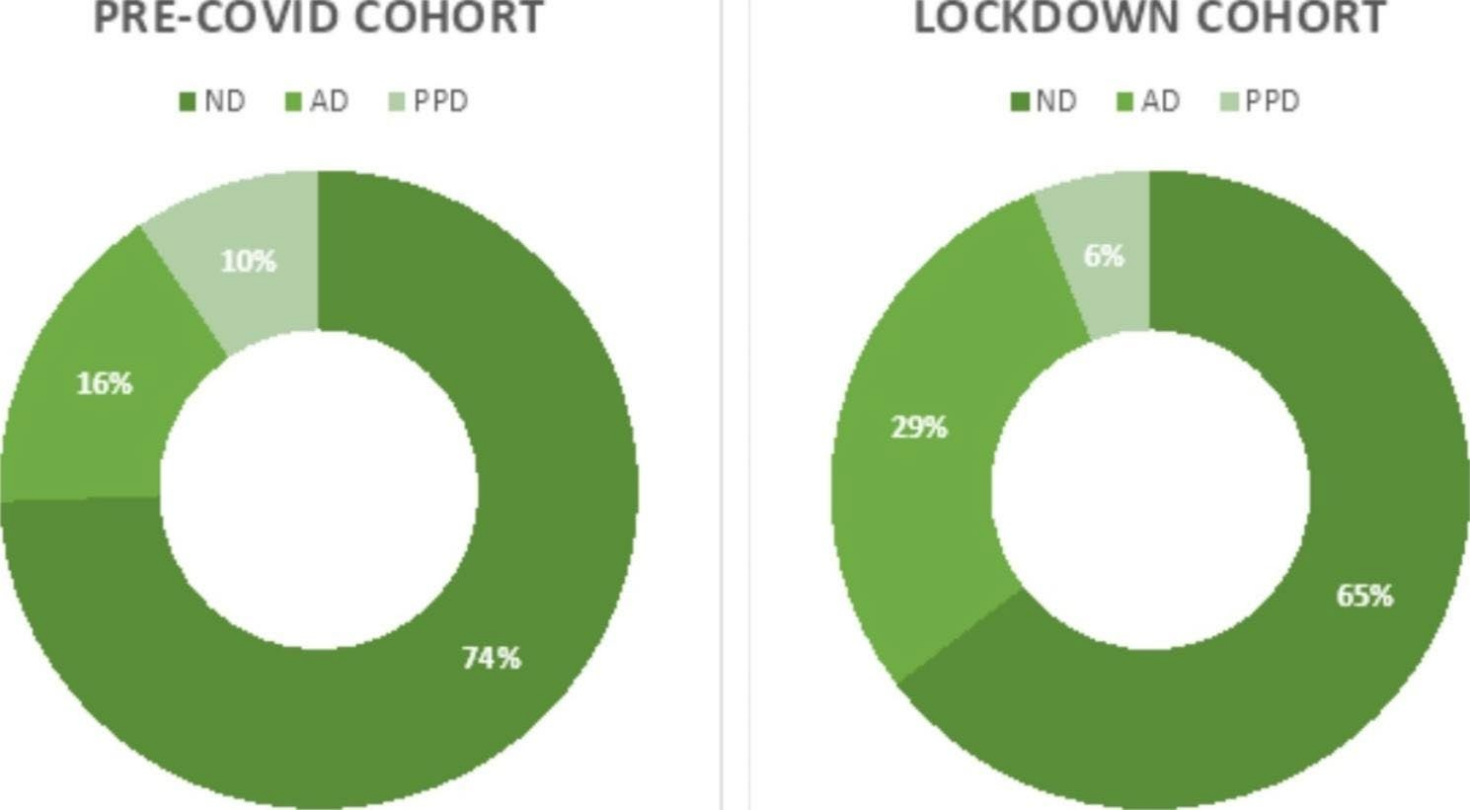
Percentage comparison of the diagnostic subgroups for both time periods Note: ND: non-depressed; AD: adjustment disorder; PPD: postpartum depression

The whole cohort consisted of 327 women. In the “pre-COVID” cohort (211 women), 16.1% participants developed AD (95% CI: 11.4–21.3%) during the postpartum follow-up, while 9.5% were diagnosed with PPD (95% CI: 5.7–13.7%). With the onset of the pandemic (“lockdown” cohort, 116 women), 29.3% (95% CI: 21.6–37.9%) qualified for an AD and 6% (95% CI: 1.7–10.3%) were diagnosed with clinical PPD. Upon comparison of CIs, no significant difference at the 95% significance level was observed between the prevalence of ND and PPD, while the prevalence of AD was significantly higher in the “lockdown” cohort with an increase of 13.2% (Fig. 2).

### Differences between “pre-COVID” and “Lockdown” cohorts (ND vs. AD vs. PPD)

Comparing the clinical-anamnestic and demographic data without a diagnostic separation between the “pre-COVID” and “lockdown” cohorts (Table 1), we found no significant differences in the anamneses of the two cohorts.

### Comparison between the diagnostic groups before and during lockdown

In terms of anamnesis, no significant differences were found in ND between the “pre-COVID” and “lockdown” cohorts (Table SM4). A slightly smaller percentage of women diagnosed with AD during the lockdown period had a psychiatric history (76.5% had no previous psychiatric diagnosis), compared to 50.0% from the “pre-COVID” cohort (p = 0.043) (SM5), although the difference was not significant under multiple corrections. Similarly, at the trend level, fewer women diagnosed with PPD during the pandemic had a family psychiatric history (p = 0.026) compared to the “pre-COVID” cohort (SM 6).

### Risk profiles associated with AD and PPD in the whole sample

Further, we investigated the differences in the clinical-anamnestic and demographic data (Table 2) associated with diagnoses in the whole cohort independent of the COVID-19 restrictions timeline. Significant differences between the subgroups were found in several cases, with a significance level of p≤0.001.

Compared to ND, more women diagnosed with PPD had a psychiatric history (51.9% in PPD vs. 15.5% in ND), had more numerous SLE (2.0±1.8 in PPD vs. 0.9±1.3 in ND), and more frequent and more severe PMS (moderate to strong PMS for 77.8% of PPD vs. 40.9% of ND) (Table SM9). The same risk factors were associated with AD (Table SM12). While AD and PPD did not differ in their risk profiles (Table SM15), a significant difference was noticed in the severity of baby blues. In the whole cohort, out of all subgroups, women with PPD reached the highest score in the MBQ (15.7±5.1), followed by AD (13.1±4.7) with ND scoring 7.9±4.3 (Table SM18).

*Differences in Depressed Mood, Mother-to-Child Attachment, Stress Scores and Temporal Trajectories between the Diagnoses in the Whole Cohort*.

**Fig. 3:**
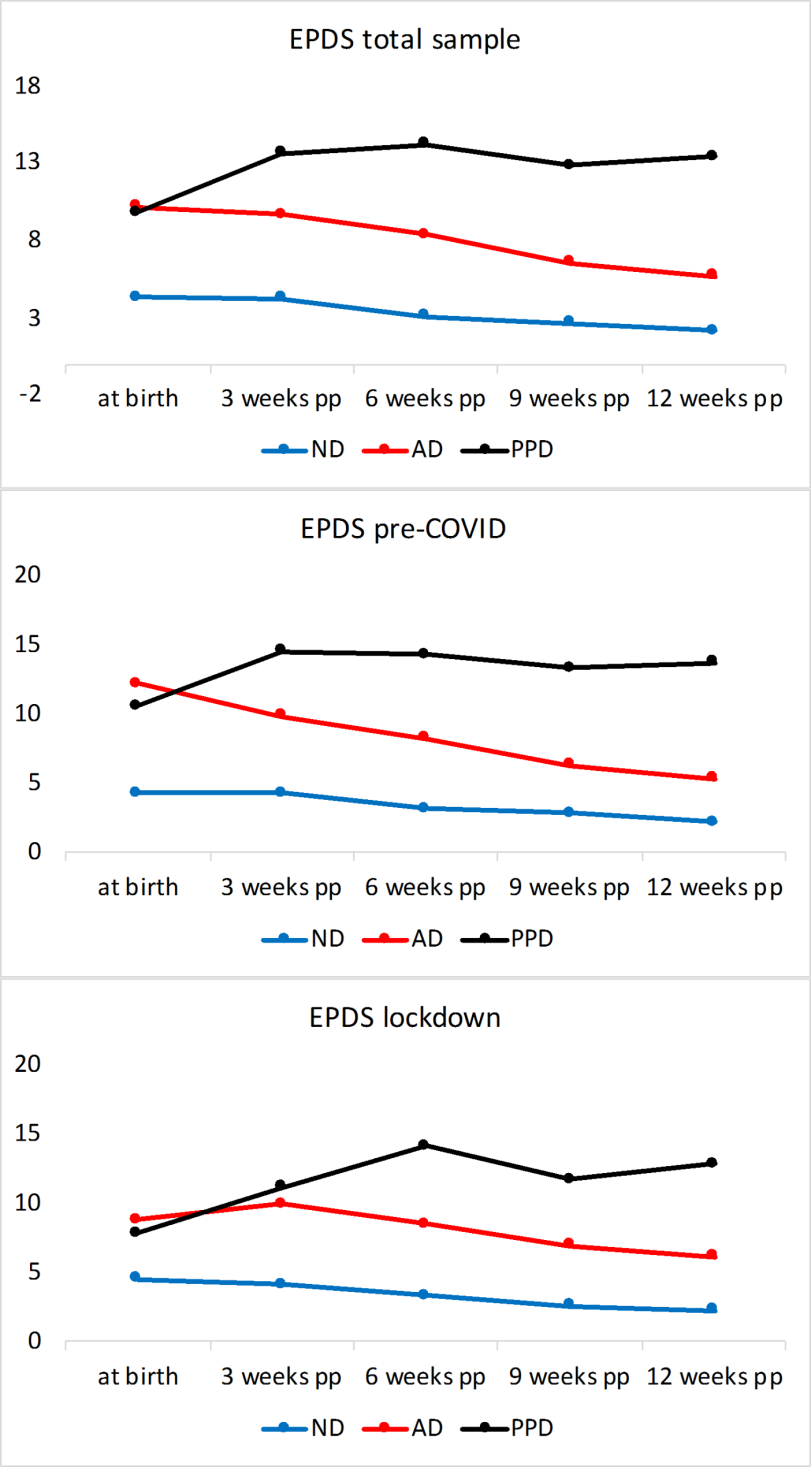
- EPDS scores across all three groups, “pre-COVID” vs. “lockdown” sample, including 95% confidence interval. Note: Pp: postpartum; ND: non-depressed; AD: adjustment disorder; PPD: postpartum depression; EPDS: Edinburgh Postpartum Depression Scale

**Fig. 4:**
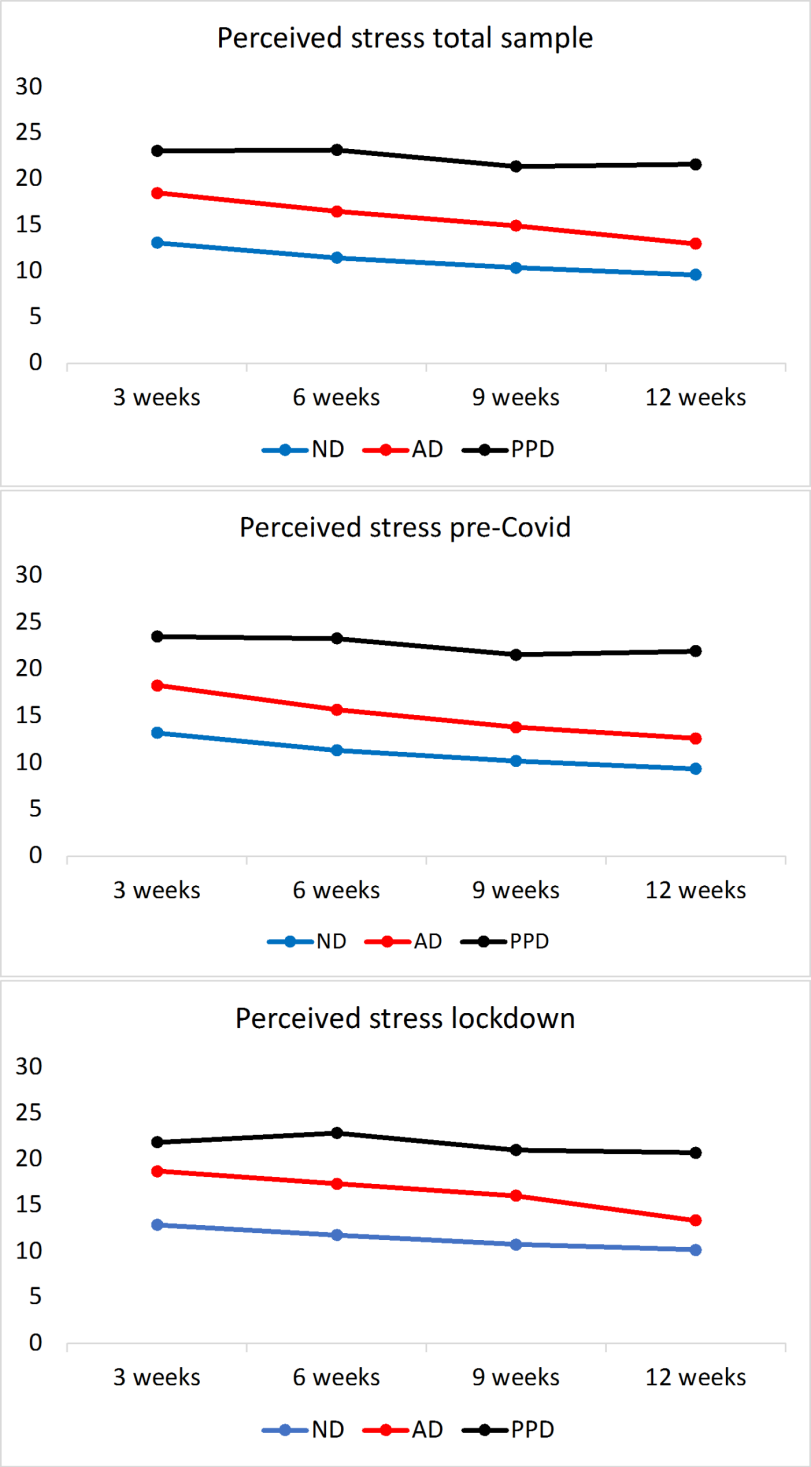
- PSS scores across all three groups, “pre-COVID” vs. “lockdown” sample, including 95% confidence interval. Note: pp: postpartum; ND: non-depressed; AD: adjustment disorder; PPD: postpartum depression; PSS: Perceived Stress Scale

**Fig. 5:**
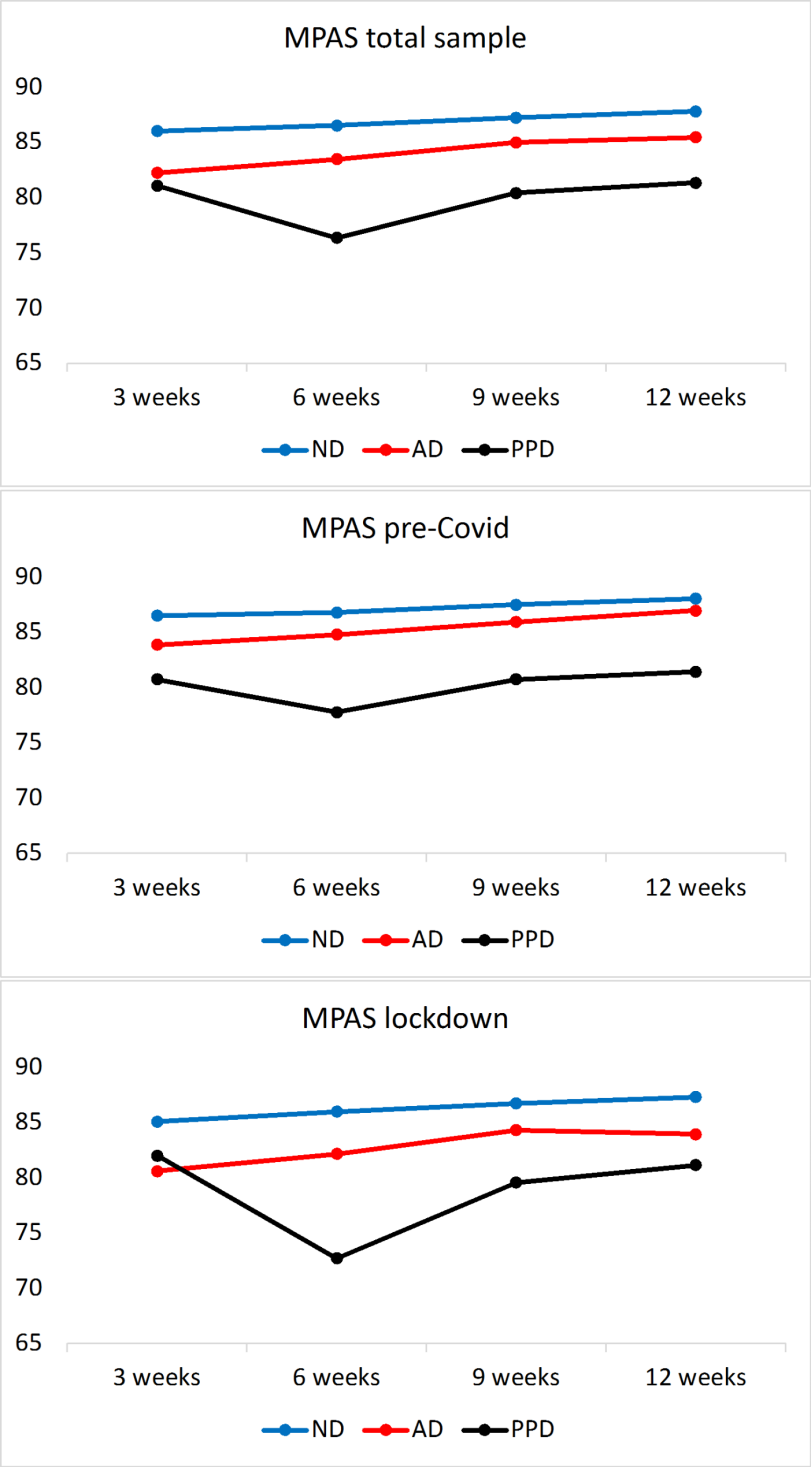
– MPAS scores across all three groups, whole sample, “pre-COVID” vs. “lockdown” sample, including 95% confidence interval. Note: pp: postpartum; ND: non-depressed; AD: adjustment disorder; PPD: postpartum depression; MPAS: Maternal Postnatal Attachment Scale

The temporal development of depressed mood, mother-to-child attachment and stress for the first 12 weeks postpartum are plotted in Figs. 3 − 5, while the p-values for the comparisons of single values are provided in Tables SM18-20. In the whole sample, depressed mood, mother-to-child attachment and stress differed significantly at all time points between AD and ND, and between PPD and ND.

The trajectories of depressed mood in ND remained consistently under 10 at all time points. PPD demonstrated an increase in depressed mood values, being as high as 9.9±4.6 already at childbirth (T0) and showing a tendency of further increase at later time points. The AD group showed a reduction from around 10 at childbirth to lower depressed mood values at subsequent time points. However, AD showed higher mood values in comparison to ND at all time points (Fig. 3) with the stress values showing a similar tendency (Fig. 4). The PPD group had significantly higher stress values at all time points in comparison to ND and AD. In AD, while the stress values decreased over time, they remained higher in comparison to ND (Fig. 4).

While in ND mother-to-child attachment remained high during the postpartum period, in AD it showed slight improvement with time, though remaining significantly lower than that of ND at all time points. In PPD, mother-to-child attachment was the lowest at T2, and only slightly improved over time. The attachment levels differed significantly among the three diagnostic groups, remaining lower in PPD compared to AD and ND at all time points.

In sum, compared to their non-depressed counterparts, women with PPD and AD demonstrated significantly higher stress, higher depressed mood and lower mother-to-child attachment throughout the postpartum period (Figs. 3, 4 and 5, Tables SM18, 21, 24). Between PPD and AD, the former had higher depressed mood scores at all time points except at T0, when they were not significantly different from those of AD. In a comparison between PPD and AD, mother-to-child attachment scores were not found to differ significantly except at T2, when PPD had lower scores (77.0±10.9). Stress was significantly higher in the PPD cohort compared to AD at all time points.

### Effect of lockdown on depressed mood, stress and attachment

We also compared the differences in the questionnaire results between the “pre-COVID” and “lockdown” cohorts (Tables SM19-20). On the whole, the trajectories looked similar to those of the whole cohort (Figs. 3, 5 and 4) with a few exceptions: Between the diagnostic subgroups in the “pre-COVID” and “lockdown” cohorts, the only significant difference in depressed mood was noted in AD at T0, with the depressed mood scores of AD in the “pre-COVID” group being significantly higher than those of the “lockdown” AD cohort (Tables SM 30–34). Nevertheless, the “lockdown” AD cohort demonstrated slightly higher stress compared to the “pre-COVID” AD group. Detailed comparisons of the psychological test scores of all diagnostic groups within the two cohorts are provided in SM4, columns “pre-COVID” cohort and “lockdown” cohort.

## Discussion

In this study, we investigated the development of PPD and AD in women after childbirth before and during the COVID-19 lockdown in Germany. We observed a 13.2% increase in the percentage of women meeting the criteria for AD in the postpartum period during the COVID-19 pandemic and lockdown (22.03.2020 – December 2020) in comparison to the baseline (November 2018 − 21.03.2020). However, contrary to what had been expected, no difference in the prevalence of PPD was observed in the “lockdown” sample. While AD typically develops as a temporary mood disorder and as a reaction to external stress factors [65], biological (i.e. hormonal) and genetic factors play a more dominant role in the development of PPD [51, 66]. Therefore, the pandemic lockdown (in the absence of other risk factors) may be viewed only as a transient stress factor, rather than an external stressor capable of triggering depression in individuals without a risk profile. The increase seen in the prevalence of AD during the pandemic might have been due to this transient stress factor [53, 67–69].

Our results contradict those of the cross-national study by Davenport et al. [35], which reported a 41% prevalence of probable perinatal and postpartum depression during the pandemic. The conclusion of this study was based on an online survey yielding an EPDS score above 13. Another study, involving Canadian women, reported 65% higher odds for developing clinically relevant postpartum depressive symptoms during the pandemic [70]. A German study found a clear association between the mental distress induced by the COVID-19 pandemic and the risk of developing postpartum depressive symptomatology [71]. However, it is conceivable that this disparity in results between our study, which did not detect any change in the PPD prevalence, and the ones mentioned above is due to the fact that in those studies, unlike in ours, no differentiation was made between AD and PPD based on a clinical interview. While the EPDS score reflects the present depressed state quite well, a clinical interview is more accurate [50, 72]. The use of only self-reported EPDS scores for grading the depressed mood might have led to the increased prevalence of PPD reported repeatedly [35, 70].

In line with our previous observations [42, 53], both PPD and AD were found to be associated with several risk factors. In particular, women with either a previous psychiatric history, experiences of stressful life events in the past, severe PMS or insufficient support at home were at a significantly heightened risk of experiencing PPD or AD in our sample. In the “pre-COVID” cohort, a psychiatric history and deficient support at home were the sole risk factors for experiencing AD. The experience of stressful life events in the past, severe PMS and insufficient support at home were related to higher odds for a PPD. In the “lockdown” sample, only a psychiatric history and insufficient support at home were identified as significant risk factors for PPD, and no significant risk factors were connected to the development of AD. Thus, fewer risk factors were found to correlate with the development of AD or PPD in the “lockdown” sample. Without further risk factors, the pandemic-related restrictions likely caused the increase seen in the prevalence of AD in new mothers, who under normal circumstances would not suffer any affective disturbance.

Investigating the lockdown’s psychological impact on the diagnostic subgroups, we compared the trajectories of depressive mood, stress and mother-child attachment between the two cohorts and found them to differ significantly independent of the COVID-19 timeline. The mood values behaved similarly across the cohorts, confirming the finding in [42]. While the depressed mood values remained consistently low in ND during the 12-week postpartum period, AD showed a decline to clinically irrelevant values, suggesting a self-remitting character of most AD cases, and PPD demonstrated an increase in the values. For ND, mother-child attachment remained high in the postpartum period, while it improved slightly over time for AD but remained lower than ND at all time points. The mother-to-child attachment for PPD was lowest at 6 weeks postpartum and improved thereafter. Women diagnosed with AD or PPD demonstrated significantly higher stress and depressed mood, and lower mother-to-child attachment as compared to ND at all time points.

The only minimal differences within the diagnoses affected by the pandemic, which however were not significant under multiple comparisons, were observed in AD. In the “lockdown” cohort, women with AD reported slightly lower mother-to-child attachment and slightly higher stress throughout the postpartum period than their counterparts in the “pre-COVID” cohort. For ND and PPD, no significant differences in depressed mood, stress and mother-to-child attachment scores were observed between the “lockdown” and “pre-COVID” cohorts. This may be another indication of the stable nature of the ND and PPD groups in the face of challenges such as the ones posed by the current circumstances. The development of PPD was found to be determined by persistent anamnestic aspects rather than temporal stressors, while ND remained at a low risk at all time points due to the absence of predisposing factors.

The restrictive measures imposed in the wake of the COVID-19 pandemic in Germany impacted many aspects of public mental health. The pandemic-related changes in some domains of everyday life might also have had a positive impact on adult mental health [21]. The findings of [20, 22] based on data from the German Health Update (GEDA 2019/2020-EHIS) describe a temporally significant decrease in depressive symptoms among German adults in the first phase of the pandemic compared to the pre-pandemic times. The overall decline in depressive symptomatology could be due partly to the pandemic-induced changes such as slow-down at work, leading to a decrease in individual stress-elicited symptoms [21] [23]., on the other hand, reported a slight increase in depressive symptoms, attributing it to financial distress. As reported by Prati and Mancini [73], the psychological impact of the pandemic-related lockdown was heterogeneous and of limited significance. Our results also indicate that, contrary to the assumptions made at the beginning of the pandemic, its impact here might have been rather mild. In 2020, Salari et al. [74] expressed serious concerns regarding the mental health of the general population during the pandemic. Focused on psychological symptoms, the meta-analysis reported a high prevalence of stress, anxiety and depression in the general population [74]. However, given the absence of control groups, the pandemic cannot be regarded as a determining factor. According to Bonanno et al. [75], most people experience a stable pattern of adaptation following a stressful event. The pandemic-related stress unfolded as a transient occurrence, lacking the severity to trigger a psychiatric condition such as depression. Moreover, the response to an acute stressor is typically contextual and depends on individual factors, rather than being a general, homogenous reaction [75]. The results of our study reaffirm the capacity for adaptation and mental resilience in postpartum women in the face of an external stressor.

However, several potential limitations of this study need to be considered. First, only German-speaking women were included in the study; therefore, the cohort was not representative of the entire maternal population in Germany with mothers from a migration background unable to participate. Also, for the most part, the participants’ levels of education ranged from average to high; thus, they were not representative of mothers with lower levels of education. In addition, the participants represented a higher socioeconomic class. Nevertheless, the study facilitated close observation of a large population of postpartum women. The depressed mood scores were actively monitored throughout the course of the study, and the mothers were contacted if their mood scores decreased. A clinical interview was conducted at the end of the study, and women in need of specialized help were provided relevant information and were referred to a specialist. The data collection procedure remained unchanged despite the circumstances involving the pandemic, allowing valid comparisons between the two cohorts.

## Conclusions

As anticipated, our data confirmed the link between psychiatric history and traumatic life experiences in the past and psychopathology in the postpartum period. Taken together, the mothers who experienced a postpartum AD during the pandemic were found to have fewer risk factors compared to their counterparts from the pre-pandemic times. Women with a history of psychiatric illness and lack of sufficient support at home remained at a significantly higher risk regardless of the COVID-19 timeline. The increase of AD in postpartum women and changes in their attachment and stress levels were likely linked to the COVID-19 crisis, indicating the role of external influences in the development and course of AD. Thus, a brief, self-limiting and subclinical deterioration in mood (AD) was observed during the lockdown, followed by a successful adaptation in most cases. On the other hand, the fact that no pandemic-induced increase was seen in clinical PPD points to a stronger than anticipated psychological resilience on the part of the new mothers.

**Table 1:** Comparison between the differences across groups without consideration of diagnosis in the “pre-COVID” and “lockdown” samples

		pre-COVID (n = 211)		Lockdown (n = 116)		P
		95%CI	%	95%CI	%	
Age		[32.06,33.35]		[32.04,33.47]		0.927
Family status	single parent		2.8		2.6	1.000
	with partner		97.2		97.4	
Marital status	unmarried		25.1		31.0	0.299
	married		74.9		69.0	
Total number of children		[1.55,1.78]		[1.44,1.69]		0.267
Degree of education	lowest		19.0		16.4	0.854
	middle		24.6		25.9	
	highest		56.4		57.8	
Annual income	< 20k		7.1		10.5	0.054
	20k-50k		35.1		23.3	
	more than 50k/year		55.9		68.1	
Complication at birth	no		70.6		75.9	0.365
	yes		29.4		24.1	
Relocation to paediatric ward	no		72.0		75.0	0.604
	yes		28.0		25.0	
Psychiatric history	no		75.4		81.0	0.271
	yes		24.6		19.0	
Familial psychiatric history	no		70.6		77.6	0.194
	yes		29.4		22.4	
Stressful life events		[1.05,1.5]		[0.72,1.2]		0.065
Support at home	very good		39.8		37.1	0.857
	good		37.9		41.4	
	satisfactory		14.2		16.4	
	sufficient		6.6		4.3	
	deficient		1.4		0.9	
PMS Severity	none/mild		51.7		50.0	0.915
	moderate		31.8		31.9	
	severe		16.6		18.1	

**Table 2:** Study population description, with groups separated based on diagnosis

		Healthy (n = 232)		Postpartum depression (n = 27)		Adjustment disorder (n = 68)		P
		95%CI	%	95%CI	%	95%CI	%	
Age		[32.4,33.49]		[29.21,34.19]		[31.28,33.44]		0.296
Family status	single parent		1.7		7.4		2.9	0.118*
	with partner		98.3		92.6		97.1	
Marital status	unmarried		25.0		33.3		33.8	0.263
	married		75.0		66.7		66.2	
Total number of children		[1.51,1.71]		[1.4,2.08]		[1.45,1.87]		0.722
Degree of education	lowest		19.4		14.8		14.7	0.621
	middle		22.0		25.9		30.9	
	highest		58.6		59.3		54.4	
Annual income	< 20k		8.2		18.5		4.4	0.019*
	20k-50k		28.0		29.6		41.2	
	more than 50k/year		63.8		51.9		54.4	
Complication at birth	No		77.2		66.7		64.7	0.076
	yes		22.8		33.3		35.3	
Relocation to paediatric ward	No		75.4		66.7		67.6	0.3
	yes		24.6		33.3		32.4	
Psychiatric history	No		84.9		48.1		63.2	< 0.001
	yes		15.1		51.9		36.8	
Familial psychiatric history	No		76.7		63.0		64.7	0.064
	Yes		23.3		37.0		35.3	
Stressful life events		[0.76,1.11]		[1.33,2.74]		[1.16,2.04]		< 0.001
Premenstrual syndrome severity	none/mild		59.1		22.2		35.3	< 0.001
	Moderate		31.5		18.5		41.2	
	Severe		9.5		59.3		23.5	
Support at home	very good		42.2		22.2		33.8	< 0.001*
	Good		42.2		33.3		30.9	
	satisfactory		12.9		18.5		19.1	
	Sufficient		1.7		18.5		14.7	
	Deficient		0.9		7.4		1.5	

* Fisher’s exact test

### Electronic supplementary material

Below is the link to the electronic supplementary material.

Supplementary Material 1

## Data Availability

All data generated or analyzed during this study are included in this published article and its supplementary information files.

## Abbreviations

PTSD: Post-traumatic stress disorder
AD: Adjustment disorder
PPD: Postpartum Depression
EPDS: Edinburgh Postnatal Depression Scale
SLE: Stressful Life Events
PMS: Premenstrual syndrome
ND: Non-depressed
SLESQ: Stressful Live Events Screening Questionnaire
MPAS: Maternal Postnatal Attachment Scale
PSS: Perceived Stress Scale
MBQ: Maternity Blues Questionnaire
CI: Confidence intervall
