# Supplementary material for: Pandemic-induced increase in adjustment disorders among postpartum women in Germany
Source: BMC Womens Health. 2023 Sep 12;23:486. doi: 10.1186/s12905-023-02638-z (PMC10498631; doi:10.1186/s12905-023-02638-z)
Supplement: Supplementary file 1 — Supplementary Material 1 [file 12905_2023_2638_MOESM1_ESM.docx]

**Supplementary information**

*SM 1: Calculated pattern of the missing data.*


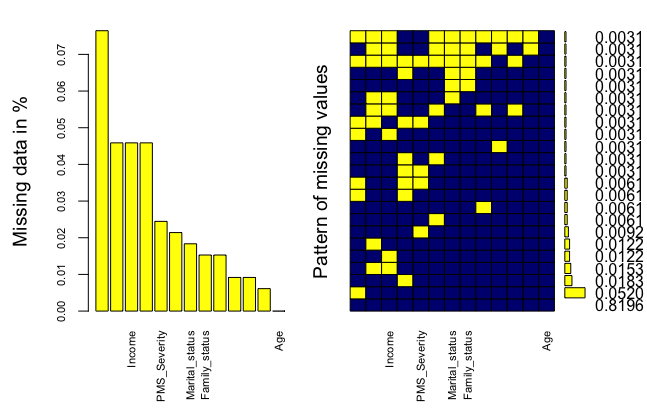


*SM 2: Differences in the full anamnesis of all three diagnostic subgroups in the “pre-COVID” sample.*

| Pre-COVID |  | **Diagnosis** |  |  |  |
| --- | --- | --- | --- | --- | --- |
|  |  | **ND (n=157)**  **n (%)** | **PPD (n=20)**  **n (%)** | **AD (n=34)**  **n (%)** | **p value** |
| Age | [95% CI] | [32.37,33.72] | [28.44,34.75] | [29.88,33.7] | 0.210 |
| Family status | single parent | 3 (1.9) | 2 (10.0) | 1 (2.9) | 0.114 |
|  | with partner | 154 (98.1) | 18 (90.0) | 33 (97.1) |  |
| Marital status | unmarried | 38 (24.2) | 7 (35.0) | 8 (23.5) | 0.576 |
|  | married | 119 (75.8) | 13 (65.0) | 26 (76.5) |  |
| Total number of children | 1 | 80 (51.0) | 11 (55.0) | 20 (58.8) | 0.466 |
|  | 2 | 54 (34.4) | 5 (25.0) | 9 (26.5) |  |
|  | 3 | 16 (10.2) | 4 (20.0) | 3 (8.8) |  |
|  | 4 | 7 (4.5) | 0 (0.0) | 1 (2.9) |  |
|  | 5 | 0 (0.0) | 0 (0.0) | 1 (2.9) |  |
| Highest degree of education | lowest | 30 (19.1) | 2 (10.0) | 8 (23.5) | 0.606 |
|  | middle | 40 (25.5) | 7 (35.0) | 6 (17.6) |  |
|  | highest | 87 (55.4) | 11 (55.0) | 20 (58.8) |  |
| Income | <20k | 13 (8.2) | 5 (25.0) | 2 (5.8) | 0.041 |
|  | 20k-50k | 49 (31.2) | 8 (40.0) | 15 (44.1) |  |
|  | more than 50k/year | 95 (60.5) | 7 (35.0) | 17 (50.0) |  |
| Complications at birth | no | 118 (75.2) | 13 (65.0) | 20 (58.8) | 0.120 |
|  | yes | 39 (24.8) | 7 (35.0) | 14 (41.2) |  |
| Relocation to another ward | no | 118 (75.2) | 13 (65.0) | 21 (61.8) | 0.222 |
|  | yes | 39 (24.8) | 7 (35.0) | 13 (38.2) |  |
| Psychiatric history | no | 132 (84.1) | 11 (55.0) | 17 (50.0) | <0.001 |
|  | yes | 25 (15.9) | 9 (45.0) | 17 (50.0) |  |
| Familial psychiatric history | no | 120 (76.4) | 10 (50.0) | 20 (58.8) | 0.012 |
|  | yes | 37 (23.6) | 10 (50.0) | 14 (41.2) |  |
| PMS Severity | none/mild | 95 (60.5) | 4 (20.0) | 11 (32.4) | <0.001 |
|  | moderate | 48 (30.6) | 4 (20.0) | 15 (44.1) |  |
|  | severe | 14 (8.9) | 12 (60.0) | 8 (23.5) |  |
| Stressful life events | 0 | 79 (50.3) | 2 (10.0) | 9 (26.5) | <0.001 |
|  | 1 | 41 (26.1) | 5 (25.0) | 8 (23.5) |  |
|  | 2 | 16 (10.2) | 7 (35.0) | 4 (11.8) |  |
|  | 3 | 9 (5.7) | 3 (15.0) | 6 (17.6) |  |
|  | 4 | 9 (5.7) | 0 (0.0) | 2 (5.9) |  |
|  | 5 | 1 (0.6) | 2 (10.0) | 2 (5.9) |  |
|  | 6 | 1 (0.6) | 0 (0.0) | 2 (5.9) |  |
|  | 7 | 0 (0.0) | 0 (0.0) | 1 (2.9) |  |
|  | 8 | 0 (0.0) | 1 (5.0) | 0 (0.0) |  |
|  | 9 | 1 (0.6) | 0 (0.0) | 0 (0.0) |  |
| Support at home | very good | 65 (41.4) | 5 (25.0) | 14 (41.2) | <0.001 |
|  | good | 68 (43.3) | 7 (35.0) | 7 (20.6) |  |
|  | satisfactory | 22 (14.0) | 2 (10.0) | 6 (17.6) |  |
|  | sufficient | 2 (1.3) | 4 (20.0) | 6 (17.6) |  |
|  | deficient | 0 (0.0) | 2 (10.0) | 1 (2.9) |  |

*SM 3: Differences in the full anamnesis of all three diagnostic subgroups in the “lockdown” sample.*

| **post COVID** |  | **Diagnosis** |  |  |  |
| --- | --- | --- | --- | --- | --- |
|  |  | **ND (n=75)**  **n (%)** | **PPD (n=7)**  **n (%)** | **AD (n=34)**  **n (%)** | **p value** |
| Age | [95% CI] | [31.8,33.69] | [27.13,36.86] | [31.83,34.04] | 0.846 |
| Family status | single parent | 2 (2.7) | 0 (0.0) | 1 (2.9) | 1.000 |
|  | with partner | 73 (97.3) | 7 (100.0) | 33 (97.1) |  |
| Marital status | unmarried | 19 (25.3) | 2 (28.6) | 15 (44.1) | 0.150 |
|  | married | 56 (74.7) | 5 (71.4) | 19 (55.9) |  |
| Total number of children | 1 | 45 (60.0) | 3 (42.9) | 17 (50.0) | 0.064 |
|  | 2 | 25 (33.3) | 1 (14.3) | 11 (32.4) |  |
|  | 3 | 5 (6.7) | 3 (42.9) | 6 (17.6) |  |
| Highest degree of education | lowest | 16 (21.3) | 2 (28.6) | 3 (8.8) | 0.067 |
|  | middle | 14 (18.7) | 0 (0.0) | 13 (38.2) |  |
|  | highest | 45 (60.0) | 5 (71.4) | 18 (52.9) |  |
| Income | <20k | 7 (9.3) | 0 (0.0) | 3 (8.8) | 0.518 |
|  | 20k-50k | 16 (21.3) | 1 (14.3) | 12 (35.3) |  |
|  | more than 50k/year | 52 (69.3) | 6 (85.7) | 19 (55.9) |  |
| Complications at birth | no | 60 (80.0) | 4 (57.1) | 24 (70.6) | 0.235 |
|  | yes | 15 (20.0) | 3 (42.9) | 10 (29.4) |  |
| Relocation to another ward | no | 56 (74.7) | 5 (71.4) | 25 (73.5) | 1.000 |
|  | yes | 19 (25.3) | 2 (28.6) | 9 (26.5) |  |
| Psychiatric history | no | 66 (88.0) | 2 (28.6) | 26 (76.5) | 0.001 |
|  | yes | 9 (12.0) | 5 (71.4) | 8 (23.5) |  |
| Familial psychiatric history | no | 59 (78.7) | 7 (100.0) | 24 (70.6) | 0.253 |
|  | yes | 16 (21.3) | 0 (0.0) | 10 (29.4) |  |
| PMS Severity | none/mild | 42 (56.0) | 3 (42.9) | 13 (38.2) | 0.095 |
|  | moderate | 24 (32.0) | 1 (14.3) | 12 (35.3) |  |
|  | severe | 9 (12.0) | 3 (42.9) | 9 (26.5) |  |
| Stressful life events | 0 | 37 (49.3) | 3 (42.9) | 17 (50.0) | 0.367 |
|  | 1 | 22 (29.3) | 0 (0.0) | 8 (23.5) |  |
|  | 2 | 6 (8.0) | 3 (42.9) | 3 (8.8) |  |
|  | 3 | 6 (8.0) | 1 (14.3) | 3 (8.8) |  |
|  | 4 | 1 (1.3) | 0 (0.0) | 2 (5.9) |  |
|  | 5 | 1 (1.3) | 0 (0.0) | 0 (0.0) |  |
|  | 6 | 2 (2.7) | 0 (0.0) | 1 (2.9) |  |
| Support at home | very good | 35 (46.7) | 1 (14.3) | 9 (26.5) | 0.001 |
|  | good | 31 (41.3) | 1 (14.3) | 14 (41.2) |  |
|  | satisfactory | 7 (9.3) | 4 (57.1) | 7 (20.6) |  |
|  | sufficient | 0 (0.0) | 1 (14.3) | 4 (11.8) |  |
|  | deficient | 2 (2.7) | 0 (0.0) | 0 (0.0) |  |

*SM 4: Overview of the significant differences in anamnesis and scores among the diagnostic groups, full cohort, “pre-COVID” and “lockdown” cohort. x- significance at the uncorrected p<0.001, * - not significant difference, but a tendency observed (p=0.005).*

|  | Full sample | | | “Pre-COVID”-cohort | | | “Lockdown”-cohort | | |
| --- | --- | --- | --- | --- | --- | --- | --- | --- | --- |
|  | ND vs AD | ND vs PPD | AD vs PPD | ND vs AD | ND vs PPD | AD vs PPD | ND vs AD | ND vs PPD | AD vs PPD |
| Previous psychiatric history | x | x |  | x | * |  |  | x |  |
| Stressful life events | x | x |  | * | x |  |  |  |  |
| Premenstrual syndrome severity | x | x |  | * | x |  |  |  |  |
| Support at home | x | x |  | x | x |  |  | x |  |
| EPDS T0 | x | x |  | x | x |  | x |  |  |
| EPDS T1 | x | x | x | x | x |  | x | x |  |
| EPDS T2 | x | x | x | x | x | x | x | x | x |
| EPDS T3 | x | x | x | x | x | x | x | x |  |
| EPDS T4 | x | x | x | x | x | x | x | x | x |
| MPAS T1 | x | x |  |  | x |  |  |  |  |
| MPAS T2 | x | x | x |  | x |  |  | x |  |
| MPAS T3 | x | x |  |  | x |  |  |  |  |
| MPAS T4 | x | x |  |  | x |  |  |  |  |
| PSS T1 | x | x | x | x | x |  | x | x |  |
| PSS T2 | x | x | x | x | x | x | x | x |  |
| PSS T3 | x | x | x | x | x | x | x | x |  |
| PSS T4 | x | x | x | x | x | x |  | x | x |
| Baby blues | x | x |  | x | x |  | x | x |  |

Note: ND: non-depressed, AD: adjustment disorder, PPD: postpartum depression, EPDS: Edinburgh Postpartum Depression Scale, MPAS: Maternal Postnatal Attachment Scale, PSS: Perceived stress scale

*SM 5: Comparison of AD women populations, “pre-COVID” vs “lockdown” cohorts.*

|  |  | **Lockdown**  **(n=34)**  **n (%)** | **pre COVID**  **(n=34)**  **n (%)** | **p** |
| --- | --- | --- | --- | --- |
| Age | [95% CI] | [31.83,34.04] | [29.88,33.7] | 0.293 |
| Family status | single parent | 1 (2.9) | 1 (2.9) | 1 |
|  | with partner | 33 (97.1) | 33 (97.1) |  |
| Marital status | unmarried | 15 (44.1) | 8 (23.5) | 0.123 |
|  | married | 19 (55.9) | 26 (76.5) |  |
| Total number of children | 1 | 17 (50.0) | 20 (58.8) | 0.512 |
|  | 2 | 11 (32.4) | 9 (26.5) |  |
|  | 3 | 6 (17.6) | 3 (8.8) |  |
|  | 4 | 0 (0.0) | 1 (2.9) |  |
|  | 5 | 0 (0.0) | 1 (2.9) |  |
| Highest degree of education | lowest | 3 (8.8) | 8 (23.5) | 0.095 |
|  | middle | 13 (38.2) | 6 (17.6) |  |
|  | highest | 18 (52.9) | 20 (58.8) |  |
| Income | <20k | 3 (8.8) | 2 (5.8) | 0.541 |
|  | 20k-50k | 12 (35.3) | 15 (44.1) |  |
|  | more than 50k/year | 19 (55.9) | 17 (50.0) |  |
| Complications at birth | no | 24 (70.6) | 20 (58.8) | 0.447 |
|  | yes | 10 (29.4) | 14 (41.2) |  |
| Relocation to another ward | no | 25 (73.5) | 21 (61.8) | 0.437 |
|  | yes | 9 (26.5) | 13 (38.2) |  |
| Psychiatric history | no | 26 (76.5) | 17 (50.0) | 0.043 |
|  | yes | 8 (23.5) | 17 (50.0) |  |
| Familial psychiatric history | no | 24 (70.6) | 20 (58.8) | 0.447 |
|  | yes | 10 (29.4) | 14 (41.2) |  |
| Stressful life events | 0 | 17 (50.0) | 9 (26.5) | 0.464 |
|  | 1 | 8 (23.5) | 8 (23.5) |  |
|  | 2 | 3 (8.8) | 4 (11.8) |  |
|  | 3 | 3 (8.8) | 6 (17.6) |  |
|  | 4 | 2 (5.9) | 2 (5.9) |  |
|  | 5 | 0 (0.0) | 2 (5.9) |  |
|  | 6 | 1 (2.9) | 2 (5.9) |  |
|  | 7 | 0 (0.0) | 1 (2.9) |  |
| Premenstrual syndrome severity | none/mild | 13 (38.2) | 11 (32.4) | 0.822 |
|  | moderate | 12 (35.3) | 15 (44.1) |  |
|  | severe | 9 (26.5) | 8 (23.5) |  |

*SM 6: Comparison of PPD women populations, “pre-COVID” vs “lockdown” cohorts.*

|  | **levels** | **lockdown**  **(n=7)**  **n (%)** | **pre COVID**  **(n=20)**  **n (%)** | **p** |
| --- | --- | --- | --- | --- |
| Age | [95% CI] | [27.13,36.86] | [28.44,34.75] | 0.888 |
| Family status | single parent | 0 (0.0) | 2 (10.0) | 1 |
|  | with partner | 7 (100.0) | 18 (90.0) |  |
| Marital status | unmarried | 2 (28.6) | 7 (35.0) | 1 |
|  | married | 5 (71.4) | 13 (65.0) |  |
| Total number of children | 1 | 3 (42.9) | 11 (55.0) | 0.516 |
|  | 2 | 1 (14.3) | 5 (25.0) |  |
|  | 3 | 3 (42.9) | 4 (20.0) |  |
| Highest degree of education | lowest | 2 (28.6) | 2 (10.0) | 0.162 |
|  | middle | 0 (0.0) | 7 (35.0) |  |
|  | highest | 5 (71.4) | 11 (55.0) |  |
| Income | <20k | 0 (0.0) | 5 (25.0) | 0.182 |
|  | 20k-50k | 1 (14.3) | 8 (40.0) |  |
|  | more than 50k/year | 6 (85.7) | 7 (35.0) |  |
| Complications at birth | no | 4 (57.1) | 13 (65.0) | 1 |
|  | yes | 3 (42.9) | 7 (35.0) |  |
| Relocation to another ward | no | 5 (71.4) | 13 (65.0) | 1 |
|  | yes | 2 (28.6) | 7 (35.0) |  |
| Psychiatric history | no | 2 (28.6) | 11 (55.0) | 0.385 |
|  | yes | 5 (71.4) | 9 (45.0) |  |
| Familial psychiatric history | no | 7 (100.0) | 10 (50.0) | 0.026 |
|  | yes | 0 (0.0) | 10 (50.0) |  |
| Stressful life events | 0 | 3 (42.9) | 2 (10.0) | 0.405 |
|  | 1 | 0 (0.0) | 5 (25.0) |  |
|  | 2 | 3 (42.9) | 7 (35.0) |  |
|  | 3 | 1 (14.3) | 3 (15.0) |  |
|  | 5 | 0 (0.0) | 2 (10.0) |  |
|  | 8 | 0 (0.0) | 1 (5.0) |  |
| Premenstrual syndrome severity | none/mild | 3 (42.9) | 4 (20.0) | 0.505 |
|  | moderate | 1 (14.3) | 4 (20.0) |  |
|  | severe | 3 (42.9) | 12 (60.0) |  |

*SM 7: Comparison of the anamnesis of ND women in the “pre- COVID” and “lockdown” samples.*

| **ND** |  | **Lockdown**  **(n=75)**  **n (%)** | **pre-COVID**  **(n=157)**  **n (%)** | **p value** |
| --- | --- | --- | --- | --- |
| Age | [95% CI] | [31.8,33.69] | [32.37,33.72] | 0.609 |
| Family status | single parent | 2 (2.7) | 3 (1.9) | 0.659 |
|  | with partner | 73 (97.3) | 154 (98.1) |  |
| Marital status | unmarried | 19 (25.3) | 38 (24.2) | 0.871 |
|  | married | 56 (74.7) | 119 (75.8) |  |
| Total number of children | 1 | 45 (60.0) | 80 (51.0) | 0.2 |
|  | 2 | 25 (33.3) | 54 (34.4) |  |
|  | 3 | 5 (6.7) | 16 (10.2) |  |
|  | 4 | 0 (0.0) | 7 (4.5) |  |
| Highest degree of education | lowest | 16 (21.3) | 30 (19.1) | 0.532 |
|  | middle | 14 (18.7) | 40 (25.5) |  |
|  | highest | 45 (60.0) | 87 (55.4) |  |
| Income | <20k | 7 (9.3) | 13 (8.2) | 0.363 |
|  | 20k-50k | 16 (21.3) | 49 (31.2) |  |
|  | more than 50k/year | 52 (69.3) | 95 (60.5) |  |
| Complications at birth | no | 60 (80.0) | 118 (75.2) | 0.507 |
|  | yes | 15 (20.0) | 39 (24.8) |  |
| Relocation to another ward | no | 56 (74.7) | 118 (75.2) | 1 |
|  | yes | 19 (25.3) | 39 (24.8) |  |
| Psychiatric history | no | 66 (88.0) | 132 (84.1) | 0.552 |
|  | yes | 9 (12.0) | 25 (15.9) |  |
| Familial psychiatric history | no | 59 (78.7) | 120 (76.4) | 0.741 |
|  | yes | 16 (21.3) | 37 (23.6) |  |
| Stressful life events | 0 | 37 (49.3) | 79 (50.3) | 0.553 |
|  | 1 | 22 (29.3) | 41 (26.1) |  |
|  | 2 | 6 (8.0) | 16 (10.2) |  |
|  | 3 | 6 (8.0) | 9 (5.7) |  |
|  | 4 | 1 (1.3) | 9 (5.7) |  |
|  | 5 | 1 (1.3) | 1 (0.6) |  |
|  | 6 | 2 (2.7) | 1 (0.6) |  |
|  | 9 | 0 (0.0) | 1 (0.6) |  |
| PMS Severity | none/mild | 42 (56.0) | 95 (60.5) | 0.676 |
|  | moderate | 24 (32.0) | 48 (30.6) |  |
|  | severe | 9 (12.0) | 14 (8.9) |  |
| Support at home | very good | 35 (46.7) | 65 (41.4) | 0.171 |
|  | good | 31 (41.3) | 68 (43.3) |  |
|  | satisfactory | 7 (9.3) | 22 (14.0) |  |
|  | sufficient | 0 (0.0) | 2 (1.3) |  |
|  | deficient | 2 (2.7) | 0 (0.0) |  |

*SM 8: Comparison between the differences across groups without consideration of diagnosis in the “pre-COVID” and “lockdown” samples.*

|  |  | **Lockdown**  **(n=116)**  **n (%)** | **pre-COVID**  **(n=211)**  **n (%)** | **p value** |
| --- | --- | --- | --- | --- |
| Age | [95% CI] | [32.04,33.47] | [32.06,33.35] | 0.927 |
| Family status | single parent | 3 (2.6) | 6 (2.8) | 1.000 |
|  | with partner | 113 (97.4) | 205 (97.2) |  |
| Marital status | unmarried | 36 (31.0) | 53 (25.1) | 0.299 |
|  | married | 80 (69.0) | 158 (74.9) |  |
| Total number of children | [95% CI] | [1,43,1.68] | [1,55,1.78] | 0.267 |
| Highest degree of education | lowest | 19 (16.4) | 40 (19.0) | 0.854 |
|  | middle | 30 (25.9) | 52 (24.6) |  |
|  | highest | 67 (57.8) | 119 (56.4) |  |
| Income | <20k | 14 (10.5) | 15 (7.1) | 0.054 |
|  | 20k-50k | 27 (23.3) | 74 (35.1) |  |
|  | more than 50k/year | 79 (68.1) | 118 (55.9) |  |
| Complications at birth | no | 88 (75.9) | 149 (70.6) | 0.365 |
|  | yes | 28 (24.1) | 62 (29.4) |  |
| Relocation to another ward | no | 87 (75.0) | 152 (72.0) | 0.604 |
|  | yes | 29 (25.0) | 59 (28.0) |  |
| Psychiatric history | no | 94 (81.0) | 159 (75.4) | 0.271 |
|  | yes | 22 (19.0) | 52 (24.6) |  |
| Familial psychiatric history | no | 90 (77.6) | 149 (70.6) | 0.194 |
|  | yes | 26 (22.4) | 62 (29.4) |  |
| Stressful life events | [95% CI] | [0,76,1,28] | [1,05,1,49] | 0.065 |
| Support at home | very good | 43 (37.1) | 84 (39.8) | 0.857 |
|  | good | 48 (41.4) | 80 (37.9) |  |
|  | satisfactory | 19 (16.4) | 30 (14.2) |  |
|  | sufficient | 5 (4.3) | 14 (6.6) |  |
|  | deficient | 1 (0.9) | 3 (1.4) |  |
| PMS Severity | none/mild | 58 (50.0) | 109 (51.7) | 0.915 |
|  | moderate | 37 (31.9) | 67 (31.8) |  |
|  | severe | 21 (18.1) | 35 (16.6) |  |
| EPDS T0 | [95% CI] | [5.15,6.54] | [5.52,6.82] | 0.527 |
| EPDS T1 | [95% CI] | [5.41,6.97] | [5.53,6.86] | 0.999 |
| EPDS T2 | [95% CI] | [4.68,6.28] | [4.31,5.58] | 0.313 |
| EPDS T3 | [95% CI] | [3.72,5.18] | [3.77,4.96] | 0.859 |
| EPDS T4 | [95% CI] | [3.32,4.82] | [3.21,4.36] | 0.547 |
| MPAS T1 | [95% CI] | [82.23,84.9] | [84.7,86.35] | 0.010 |
| MPAS T2 | [95% CI] | [82.67,85.39] | [84.8,86.53] | 0.035 |
| MPAS T3 | [95% CI] | [84.31,86.7] | [85.86,87.33] | 0.102 |
| MPAS T4 | [95% CI] | [84.86,87.01] | [86.51,87.98] | 0.041 |
| Stress T1 | [95% CI] | [13.98,16.27] | [14.12,15.86] | 0.855 |
| Stress T2 | [95% CI] | [12.97,15.18] | [12.2,13.86] | 0.136 |
| Stress T3 | [95% CI] | [11.82,14.03] | [10.88,12.54] | 0.079 |
| Stress T4 | [95% CI] | [10.70,12.76] | [10.22,11.89] | 0.326 |
| baby blues | [95% CI] | [8.92,10.81] | [8.71,10.17] | 0.487 |
| Diagnose | ND | 75 (64.7) | 157 (74.4) | 0.018 |
|  | PPD | 7 (6.0) | 20 (9.5) |  |
|  | AD | 34 (29.3) | 34 (16.1) |  |

*SM 9: Comparison of the anamnesis of ND and PPD in the full cohort.*

| **label** | **levels** | **ND**  **(n=232)**  **n (%)** | **PPD**  **(n=27)**  **n (%)** | **p value** |
| --- | --- | --- | --- | --- |
| Age | [95% CI] | [32.4,33.49] | [29.21,34.19] | 0.171 |
| Family status | single parent | 4 (1.7) | 2 (7.4) | 0.121 |
|  | with partner | 228 (98.3) | 25 (92.6) |  |
| Marital status | unmarried | 57 (24.6) | 9 (33.3) | 0.352 |
|  | married | 175 (75.4) | 18 (66.7) |  |
| Total number of children | [95% CI] | [1.51,1.71] | [1.4,2.08] | 0.44 |
| Highest degree of education | lowest | 47 (20.3) | 4 (14.8) | 0.788 |
|  | middle | 53 (22.8) | 7 (25.9) |  |
|  | highest | 132 (56.9) | 16 (59.3) |  |
| Income | <20k | 18 (7.7) | 6 (22.2) | 0.045 |
|  | 20k-50k | 67 (28.9) | 8 (29.6) |  |
|  | more than 50k/year | 147 (63.4) | 13 (48.1) |  |
| Complications at birth | no | 179 (77.2) | 15 (55.6) | 0.019 |
|  | yes | 53 (22.8) | 12 (44.4) |  |
| Relocation to another ward | no | 175 (75.4) | 18 (66.7) | 0.352 |
|  | yes | 57 (24.6) | 9 (33.3) |  |
| Psychiatric history | no | 196 (84.5) | 13 (48.1) | <0.001 |
|  | yes | 36 (15.5) | 14 (51.9) |  |
| Familial psychiatric history | no | 178 (76.7) | 17 (63.0) | 0.155 |
|  | yes | 54 (23.3) | 10 (37.0) |  |
| Stressful life events | [95% CI] | [0.76,1.11] | [1.33,2.74] | <0.001 |
| PMS Severity | none/mild | 137 (59.1) | 6 (22.2) | <0.001 |
|  | moderate | 72 (31.0) | 6 (22.2) |  |
|  | severe | 23 (9.9) | 15 (55.6) |  |
| Support at home | very good | 102 (44.0) | 5 (18.5) | <0.001 |
|  | good | 98 (42.2) | 8 (29.6) |  |
|  | satisfactory | 29 (12.5) | 7 (25.9) |  |
|  | sufficient | 2 (0.9) | 5 (18.5) |  |
|  | deficient | 1 (0.4) | 2 (7.4) |  |

*SM 10: Comparison of the anamnesis of ND and PPD in the “pre-COVID” cohort.*

| **label** | **levels** | **ND**  **(n=157)**  **n (%)** | **PPD**  **(n=20)**  **n (%)** | **p value** |
| --- | --- | --- | --- | --- |
| Age | [95% CI] | [32.37,33.72] | [28.44,34.75] | 0.187 |
| Family status | single parent | 3 (1.9) | 2 (10.0) | 0.099 |
|  | with partner | 154 (98.1) | 18 (90.0) |  |
| Marital status | unmarried | 38 (24.2) | 7 (35.0) | 0.288 |
|  | married | 119 (75.8) | 13 (65.0) |  |
| Total number of children | 1 | 80 (51.0) | 11 (55.0) | 0.476 |
|  | 2 | 54 (34.4) | 5 (25.0) |  |
|  | 3 | 16 (10.2) | 4 (20.0) |  |
|  | 4 | 7 (4.5) |  |  |
| Highest degree of education | lowest | 30 (19.1) | 2 (10.0) | 0.524 |
|  | middle | 40 (25.5) | 7 (35.0) |  |
|  | highest | 87 (55.4) | 11 (55.0) |  |
| Income | <20k | 13 (8.2) | 5 (25.0) | 0.034 |
|  | 20k-50k | 49 (31.2) | 8 (40.0) |  |
|  | more than 50k/year | 95 (60.5) | 7 (35.0) |  |
| Complications at birth | no | 118 (75.2) | 13 (65.0) | 0.416 |
|  | yes | 39 (24.8) | 7 (35.0) |  |
| Relocation to another ward | no | 118 (75.2) | 13 (65.0) | 0.416 |
|  | yes | 39 (24.8) | 7 (35.0) |  |
| Psychiatric history | no | 132 (84.1) | 11 (55.0) | 0.005 |
|  | yes | 25 (15.9) | 9 (45.0) |  |
| Familial psychiatric history | no | 120 (76.4) | 10 (50.0) | 0.017 |
|  | yes | 37 (23.6) | 10 (50.0) |  |
| Stressful life events | 0 | 79 (50.3) | 2 (10.0) | <0.001 |
|  | 1 | 41 (26.1) | 5 (25.0) |  |
|  | 2 | 16 (10.2) | 7 (35.0) |  |
|  | 3 | 9 (5.7) | 3 (15.0) |  |
|  | 4 | 9 (5.7) |  |  |
|  | 5 | 1 (0.6) | 2 (10.0) |  |
|  | 6 | 1 (0.6) |  |  |
|  | 9 | 1 (0.6) |  |  |
|  | 8 |  | 1 (5.0) |  |
| PMS Severity | none/mild | 95 (60.5) | 4 (20.0) | <0.001 |
|  | moderate | 48 (30.6) | 4 (20.0) |  |
|  | severe | 14 (8.9) | 12 (60.0) |  |
| Support at home | very good | 65 (41.4) | 5 (25.0) | <0.001 |
|  | good | 68 (43.3) | 7 (35.0) |  |
|  | satisfactory | 22 (14.0) | 2 (10.0) |  |
|  | sufficient | 2 (1.3) | 4 (20.0) |  |
|  | deficient |  | 2 (10.0) |  |

*SM 11: Comparison of the anamnesis of ND and PPD in the “lockdown” cohort.*

| **label** | **levels** | **ND**  **(n=75)**  **n (%)** | **PPD**  **(n=7)**  **n (%)** | **p value** |
| --- | --- | --- | --- | --- |
| Age | [95% CI] | [31.8,33.69] | [27.13,36.86] | 0.654 |
| Family status | single parent | 2 (2.7) | 0 (0.0) | 1 |
|  | with partner | 73 (97.3) | 7 (100.0) |  |
| Marital status | unmarried | 19 (25.3) | 2 (28.6) | 1 |
|  | married | 56 (74.7) | 5 (71.4) |  |
| Total number of children | 1 | 45 (60.0) | 3 (42.9) | 0.028 |
|  | 2 | 25 (33.3) | 1 (14.3) |  |
|  | 3 | 5 (6.7) | 3 (42.9) |  |
| Highest degree of education | lowest | 16 (21.3) | 2 (28.6) | 0.631 |
|  | middle | 14 (18.7) | 0 (0.0) |  |
|  | highest | 45 (60.0) | 5 (71.4) |  |
| Income | <20k | 7 (9.3) | 0 (0.0) | 1 |
|  | 20k-50k | 16 (21.3) | 1 (14.3) |  |
|  | more than 50k/year | 52 (69.3) | 6 (85.7) |  |
| Complications at birth | no | 60 (80.0) | 4 (57.1) | 0.175 |
|  | yes | 15 (20.0) | 3 (42.9) |  |
| Relocation to another ward | no | 56 (74.7) | 5 (71.4) | 1 |
|  | yes | 19 (25.3) | 2 (28.6) |  |
| Psychiatric history | no | 66 (88.0) | 2 (28.6) | 0.001 |
|  | yes | 9 (12.0) | 5 (71.4) |  |
| Familial psychiatric history | no | 59 (78.7) | 7 (100.0) | 0.336 |
|  | yes | 16 (21.3) | 0 (0.0) |  |
| Stressful life events | 0 | 37 (49.3) | 3 (42.9) | 0.105 |
|  | 1 | 22 (29.3) | 0 (0.0) |  |
|  | 2 | 6 (8.0) | 3 (42.9) |  |
|  | 3 | 6 (8.0) | 1 (14.3) |  |
|  | 4 | 1 (1.3) | 0 (0.0) |  |
|  | 5 | 1 (1.3) | 0 (0.0) |  |
|  | 6 | 2 (2.7) | 0 (0.0) |  |
| PMS Severity | none/mild | 42 (56.0) | 3 (42.9) | 0.108 |
|  | moderate | 24 (32.0) | 1 (14.3) |  |
|  | severe | 9 (12.0) | 3 (42.9) |  |
| Support at home | very good | 35 (46.7) | 1 (14.3) | <0.001 |
|  | good | 31 (41.3) | 1 (14.3) |  |
|  | satisfactory | 7 (9.3) | 4 (57.1) |  |
|  | sufficient | 0 (0.0) | 1 (14.3) |  |
|  | deficient | 2 (2.7) | 0 (0.0) |  |

*SM 12: Comparison of the anamnesis of ND and AD in the full cohort.*

| **label** | **levels** | **ND**  **(n=232)**  **n (%)** | **AD**  **(n=68)**  **n (%)** | **p value** |
| --- | --- | --- | --- | --- |
| Age | [95% CI] | [32.4,33.49] | [31.28,33.44] | 0.322 |
| Family status | single parent | 4 (1.7) | 2 (2.9) | 0.621 |
|  | with partner | 228 (98.3) | 66 (97.1) |  |
| Marital status | unmarried | 57 (24.6) | 22 (32.4) | 0.212 |
|  | married | 175 (75.4) | 46 (67.6) |  |
| Total number of children | [95% CI] | [1.51,1.71] | [1.45,1.87] | 0.688 |
| Highest degree of education | lowest | 47 (20.3) | 10 (14.7) | 0.503 |
|  | middle | 53 (22.8) | 19 (27.9) |  |
|  | highest | 132 (56.9) | 39 (57.4) |  |
| Income | <20k | 18 (7.7) | 4 (5.9) | 0.4 |
|  | 20k-50k | 67 (28.9) | 27 (39.7) |  |
|  | more than 50k/year | 147 (63.4) | 37 (54.4) |  |
| Complications at birth | no | 179 (77.2) | 44 (64.7) | 0.057 |
|  | yes | 53 (22.8) | 24 (35.3) |  |
| Relocation to another ward | no | 175 (75.4) | 46 (67.6) | 0.212 |
|  | yes | 57 (24.6) | 22 (32.4) |  |
| Psychiatric history | no | 196 (84.5) | 43 (63.2) | <0.001 |
|  | yes | 36 (15.5) | 25 (36.8) |  |
| Familial psychiatric history | no | 178 (76.7) | 44 (64.7) | 0.059 |
|  | yes | 54 (23.3) | 24 (35.3) |  |
| Stressful life events | [95% CI] | [0.76,1.11] | [1.16,2.04] | 0.001 |
| PMS Severity | none/mild | 137 (59.1) | 24 (35.3) | <0.001 |
|  | moderate | 72 (31.0) | 27 (39.7) |  |
|  | severe | 23 (9.9) | 17 (25.0) |  |
| Support at home | very good | 102 (44.0) | 22 (32.4) | <0.001 |
|  | good | 98 (42.2) | 21 (30.9) |  |
|  | satisfactory | 29 (12.5) | 14 (20.6) |  |
|  | sufficient | 2 (0.9) | 10 (14.7) |  |
|  | deficient | 1 (0.4) | 1 (1.5) |  |

*SM 13: Comparison of the anamnesis of ND and AD in the “pre-COVID” cohort.*

| **label** | **levels** | **ND**  **(n=157)**  **n (%)** | **AD**  **(n=34)**  **n (%)** | **p value** |
| --- | --- | --- | --- | --- |
| Age | [95% CI] | [32.37,33.72] | [29.88,33.7] | 0.143 |
| Family status | single parent | 3 (1.9) | 1 (2.9) | 0.547 |
|  | with partner | 154 (98.1) | 33 (97.1) |  |
| Marital status | unmarried | 38 (24.2) | 8 (23.5) | 1 |
|  | married | 119 (75.8) | 26 (76.5) |  |
| Total number of children | 1 | 80 (51.0) | 20 (58.8) | 0.352 |
|  | 2 | 54 (34.4) | 9 (26.5) |  |
|  | 3 | 16 (10.2) | 3 (8.8) |  |
|  | 4 | 7 (4.5) | 1 (2.9) |  |
|  | 5 |  | 1 (2.9) |  |
| Highest degree of education | lowest | 30 (19.1) | 8 (23.5) | 0.599 |
|  | middle | 40 (25.5) | 6 (17.6) |  |
|  | highest | 87 (55.4) | 20 (58.8) |  |
| Income | <20k | 13 (8.2) | 2 (5.8) | 0.19 |
|  | 20k-50k | 49 (31.2) | 15 (44.1) |  |
|  | more than 50k/year | 95 (60.5) | 17 (50.0) |  |
| Complications at birth | no | 118 (75.2) | 20 (58.8) | 0.06 |
|  | yes | 39 (24.8) | 14 (41.2) |  |
| Relocation to another ward | no | 118 (75.2) | 21 (61.8) | 0.137 |
|  | yes | 39 (24.8) | 13 (38.2) |  |
| Psychiatric history | no | 132 (84.1) | 17 (50.0) | <0.001 |
|  | yes | 25 (15.9) | 17 (50.0) |  |
| Familial psychiatric history | no | 120 (76.4) | 20 (58.8) | 0.053 |
|  | yes | 37 (23.6) | 14 (41.2) |  |
| Stressful life events | 0 | 79 (50.3) | 9 (26.5) | 0.003 |
|  | 1 | 41 (26.1) | 8 (23.5) |  |
|  | 2 | 16 (10.2) | 4 (11.8) |  |
|  | 3 | 9 (5.7) | 6 (17.6) |  |
|  | 4 | 9 (5.7) | 2 (5.9) |  |
|  | 5 | 1 (0.6) | 2 (5.9) |  |
|  | 6 | 1 (0.6) | 2 (5.9) |  |
|  | 9 | 1 (0.6) |  |  |
|  | 7 |  | 1 (2.9) |  |
| PMS Severity | none/mild | 95 (60.5) | 11 (32.4) | 0.004 |
|  | moderate | 48 (30.6) | 15 (44.1) |  |
|  | severe | 14 (8.9) | 8 (23.5) |  |
| Support at home | very good | 65 (41.4) | 14 (41.2) | <0.001 |
|  | good | 68 (43.3) | 7 (20.6) |  |
|  | satisfactory | 22 (14.0) | 6 (17.6) |  |
|  | sufficient | 2 (1.3) | 6 (17.6) |  |
|  | deficient |  | 1 (2.9) |  |

*SM 14: Comparison of the anamnesis of ND and AD in the “lockdown” cohort.*

| **label** | **levels** | **ND**  **(n=75)**  **n (%)** | **AD**  **(n=34)**  **n (%)** | **p value** |
| --- | --- | --- | --- | --- |
| Age | [95% CI] | [31.8,33.69] | [31.83,34.04] | 0.807 |
| Family status | single parent | 2 (2.7) | 1 (2.9) | 1 |
|  | with partner | 73 (97.3) | 33 (97.1) |  |
| Marital status | unmarried | 19 (25.3) | 15 (44.1) | 0.073 |
|  | married | 56 (74.7) | 19 (55.9) |  |
| Total number of children | 1 | 45 (60.0) | 17 (50.0) | 0.212 |
|  | 2 | 25 (33.3) | 11 (32.4) |  |
|  | 3 | 5 (6.7) | 6 (17.6) |  |
| Highest degree of education | lowest | 16 (21.3) | 3 (8.8) | 0.058 |
|  | middle | 14 (18.7) | 13 (38.2) |  |
|  | highest | 45 (60.0) | 18 (52.9) |  |
| Income | <20k | 7 (9.3) | 3 (8.8) | 0.309 |
|  | 20k-50k | 16 (21.3) | 12 (35.3) |  |
|  | more than 50k/year | 52 (69.3) | 19 (55.9) |  |
| Complications at birth | no | 60 (80.0) | 24 (70.6) | 0.328 |
|  | yes | 15 (20.0) | 10 (29.4) |  |
| Relocation to another ward | no | 56 (74.7) | 25 (73.5) | 1 |
|  | yes | 19 (25.3) | 9 (26.5) |  |
| Psychiatric history | no | 66 (88.0) | 26 (76.5) | 0.156 |
|  | yes | 9 (12.0) | 8 (23.5) |  |
| Familial psychiatric history | no | 59 (78.7) | 24 (70.6) | 0.467 |
|  | yes | 16 (21.3) | 10 (29.4) |  |
| Stressful life events | 0 | 37 (49.3) | 17 (50.0) | 0.877 |
|  | 1 | 22 (29.3) | 8 (23.5) |  |
|  | 2 | 6 (8.0) | 3 (8.8) |  |
|  | 3 | 6 (8.0) | 3 (8.8) |  |
|  | 4 | 1 (1.3) | 2 (5.9) |  |
|  | 5 | 1 (1.3) |  |  |
|  | 6 | 2 (2.7) | 1 (2.9) |  |
| PMS Severity | none/mild | 42 (56.0) | 13 (38.2) | 0.102 |
|  | moderate | 24 (32.0) | 12 (35.3) |  |
|  | severe | 9 (12.0) | 9 (26.5) |  |
| Support at home | very good | 35 (46.7) | 9 (26.5) | 0.006 |
|  | good | 31 (41.3) | 14 (41.2) |  |
|  | satisfactory | 7 (9.3) | 7 (20.6) |  |
|  | deficient | 2 (2.7) |  |  |
|  | sufficient |  | 4 (11.8) |  |

*SM 15: Comparison of the anamnesis of PPD and AD in the full cohort.*

| **label** | **levels** | **PPD**  **(n=27)**  **n (%)** | **AD**  **(n=68)**  **n (%)** | **p value** |
| --- | --- | --- | --- | --- |
| Age | [95% CI] | [29.21,34.19] | [31.28,33.44] | 0.564 |
| Family status | single parent | 2 (7.4) | 2 (2.9) | 0.319 |
|  | with partner | 25 (92.6) | 66 (97.1) |  |
| Marital status | unmarried | 9 (33.3) | 22 (32.4) | 1 |
|  | married | 18 (66.7) | 46 (67.6) |  |
| Total number of children | [95% CI] | [1.4,2.08] | [1.45,1.87] | 0.691 |
| Highest degree of education | lowest | 4 (14.8) | 10 (14.7) | 1 |
|  | middle | 7 (25.9) | 19 (27.9) |  |
|  | highest | 16 (59.3) | 39 (57.4) |  |
| Income | <20k | 6 (22.2) | 4 (5.9) | 0.088 |
|  | 20k-50k | 8 (29.6) | 27 (39.7) |  |
|  | more than 50k/year | 13 (48.1) | 37 (54.4) |  |
| Complications at birth | no | 15 (55.6) | 44 (64.7) | 0.484 |
|  | yes | 12 (44.4) | 24 (35.3) |  |
| Relocation to another ward | no | 18 (66.7) | 46 (67.6) | 1 |
|  | yes | 9 (33.3) | 22 (32.4) |  |
| Psychiatric history | no | 13 (48.1) | 43 (63.2) | 0.248 |
|  | yes | 14 (51.9) | 25 (36.8) |  |
| Familial psychiatric history | no | 17 (63.0) | 44 (64.7) | 1 |
|  | yes | 10 (37.0) | 24 (35.3) |  |
| Stressful life events | [95% CI] | [1.33,2.74] | [1.16,2.04] | 0.298 |
| PMS Severity | none/mild | 6 (22.2) | 24 (35.3) | 0.022 |
|  | moderate | 6 (22.2) | 27 (39.7) |  |
|  | severe | 15 (55.6) | 17 (25.0) |  |
| Support at home | very good | 5 (18.5) | 22 (32.4) | 0.420 |
|  | good | 8 (29.6) | 21 (30.9) |  |
|  | satisfactory | 7 (25.9) | 14 (20.6) |  |
|  | sufficient | 5 (18.5) | 10 (14.7) |  |
|  | deficient | 2 (7.4) | 1 (1.5) |  |

*SM 16: Comparison of the anamnesis of PPD and AD in the “pre-COVID” cohort.*

| **label** | **levels** | **PPD**  **(n=20)**  **n (%)** | **AD**  **(n=34)**  **n (%)** | **p value** |
| --- | --- | --- | --- | --- |
| Age | [95% CI] | [28.44,34.75] | [29.88,33.7] | 0.908 |
| Family status | single parent | 2 (10.0) | 1 (2.9) | 0.548 |
|  | with partner | 18 (90.0) | 33 (97.1) |  |
| Marital status | unmarried | 7 (35.0) | 8 (23.5) | 0.53 |
|  | married | 13 (65.0) | 26 (76.5) |  |
| Total number of children | 1 | 11 (55.0) | 20 (58.8) | 0.845 |
|  | 2 | 5 (25.0) | 9 (26.5) |  |
|  | 3 | 4 (20.0) | 3 (8.8) |  |
|  | 4 |  | 1 (2.9) |  |
|  | 5 |  | 1 (2.9) |  |
| Highest degree of education | lowest | 2 (10.0) | 8 (23.5) | 0.249 |
|  | middle | 7 (35.0) | 6 (17.6) |  |
|  | highest | 11 (55.0) | 20 (58.8) |  |
| Income | <20k | 5 (25.0) | 2 (5.8) | 0.162 |
|  | 20k-50k | 8 (40.0) | 15 (44.1) |  |
|  | more than 50k/year | 7 (35.0) | 17 (50.0) |  |
| Complications at birth | no | 13 (65.0) | 20 (58.8) | 0.775 |
|  | yes | 7 (35.0) | 14 (41.2) |  |
| Relocation to another ward | no | 13 (65.0) | 21 (61.8) | 1 |
|  | yes | 7 (35.0) | 13 (38.2) |  |
| Psychiatric history | no | 11 (55.0) | 17 (50.0) | 0.783 |
|  | yes | 9 (45.0) | 17 (50.0) |  |
| Familial psychiatric history | no | 10 (50.0) | 20 (58.8) | 0.58 |
|  | yes | 10 (50.0) | 14 (41.2) |  |
| Stressful life events | 0 | 2 (10.0) | 9 (26.5) | 0.29 |
|  | 1 | 5 (25.0) | 8 (23.5) |  |
|  | 2 | 7 (35.0) | 4 (11.8) |  |
|  | 3 | 3 (15.0) | 6 (17.6) |  |
|  | 5 | 2 (10.0) | 2 (5.9) |  |
|  | 8 | 1 (5.0) |  |  |
|  | 4 |  | 2 (5.9) |  |
|  | 6 |  | 2 (5.9) |  |
|  | 7 |  | 1 (2.9) |  |
| PMS Severity | none/mild | 4 (20.0) | 11 (32.4) | 0.032 |
|  | moderate | 4 (20.0) | 15 (44.1) |  |
|  | severe | 12 (60.0) | 8 (23.5) |  |
| Support at home | very good | 5 (25.0) | 14 (41.2) | 0.461 |
|  | good | 7 (35.0) | 7 (20.6) |  |
|  | satisfactory | 2 (10.0) | 6 (17.6) |  |
|  | sufficient | 4 (20.0) | 6 (17.6) |  |
|  | deficient | 2 (10.0) | 1 (2.9) |  |

*SM 17: Comparison of the anamnesis of PPD and AD in the “lockdown” cohort.*

| **label** | **levels** | **PPD**  **(n=7)**  **n (%)** | **AD**  **(n=34)**  **n (%)** | **p value** |
| --- | --- | --- | --- | --- |
| Age | [95% CI] | [27.13,36.86] | [31.83,34.04] | 0.529 |
| Family status | with partner | 7 (100.0) | 33 (97.1) | 1 |
|  | single parent |  | 1 (2.9) |  |
| Marital status | unmarried | 2 (28.6) | 15 (44.1) | 0.679 |
|  | married | 5 (71.4) | 19 (55.9) |  |
| Total number of children | 1 | 3 (42.9) | 17 (50.0) | 0.417 |
|  | 2 | 1 (14.3) | 11 (32.4) |  |
|  | 3 | 3 (42.9) | 6 (17.6) |  |
| Highest degree of education | lowest | 2 (28.6) | 3 (8.8) | 0.085 |
|  | highest | 5 (71.4) | 18 (52.9) |  |
|  | middle |  | 13 (38.2) |  |
| Income | <20k |  | 3 (8.8) | 0.523 |
|  | 20k-50k | 1 (14.3) | 12 (35.3) |  |
|  | more than 50k/year | 6 (85.7) | 19 (55.9) |  |
| Complications at birth | no | 4 (57.1) | 24 (70.6) | 0.659 |
|  | yes | 3 (42.9) | 10 (29.4) |  |
| Relocation to another ward | no | 5 (71.4) | 25 (73.5) | 1 |
|  | yes | 2 (28.6) | 9 (26.5) |  |
| Psychiatric history | no | 2 (28.6) | 26 (76.5) | 0.024 |
|  | yes | 5 (71.4) | 8 (23.5) |  |
| Familial psychiatric history | no | 7 (100.0) | 24 (70.6) | 0.164 |
|  | yes |  | 10 (29.4) |  |
| Stressful life events | 0 | 3 (42.9) | 17 (50.0) | 0.207 |
|  | 2 | 3 (42.9) | 3 (8.8) |  |
|  | 3 | 1 (14.3) | 3 (8.8) |  |
|  | 1 |  | 8 (23.5) |  |
|  | 4 |  | 2 (5.9) |  |
|  | 6 |  | 1 (2.9) |  |
| PMS Severity | none/mild | 3 (42.9) | 13 (38.2) | 0.518 |
|  | moderate | 1 (14.3) | 12 (35.3) |  |
|  | severe | 3 (42.9) | 9 (26.5) |  |
| Support at home | very good | 1 (14.3) | 9 (26.5) | 0.219 |
|  | good | 1 (14.3) | 14 (41.2) |  |
|  | satisfactory | 4 (57.1) | 7 (20.6) |  |
|  | sufficient | 1 (14.3) | 4 (11.8) |  |

*SM 18: Comparison of the results from the questionnaires of all three diagnostic subgroups in the full cohort.*

|  |  | **Diagnosis** |  |  |  |
| --- | --- | --- | --- | --- | --- |
| **label** |  | **ND**  **(n=232)** | **PPD**  **(n=27)** | **AD**  **(n=68)** | **p value** |
| EPDS T0 | [95% CI] | [4.01,4.78] | [8.06,11.71] | [8.98,11.42] | <0.001 |
| EPDS T1 | [95% CI] | [3.92,4.65] | [11.21,16.11] | [8.8,10.69] | <0.001 |
| EPDS T2 | [95% CI] | [2.85,3.54] | [11.73,15.39] | [7.4,9.38] | <0.001 |
| EPDS T3 | [95% CI] | [2.44,3.14] | [10.76,14.37] | [5.74,7.54] | <0.001 |
| EPDS T4 | [95% CI] | [1.95,2.57] | [11.56,14.72] | [4.92,6.6] | <0.001 |
| MPAS T1 | [95% CI] | [85.31,86.75] | [77.72,84.42] | [80.39,84.07] | <0.001 |
| MPAS T2 | [95% CI] | [85.81,87.21] | [72.70,81.31] | [81.93,85.00] | <0.001 |
| MPAS T3 | [95% CI] | [86.58,87.85] | [76.90,84.36] | [83.67,86.29] | <0.001 |
| MPAS T4 | [95% CI] | [87.17,88.39] | [78.3,84.78] | [84.07,86.83] | <0.001 |
| Stress T1 | [95% CI] | [12.4,13.78] | [20.44,25.7] | [17.19,19.8] | <0.001 |
| Stress T2 | [95% CI] | [10.84,12.12] | [19.37,24.79] | [15.32,17.7] | <0.001 |
| Stress T3 | [95% CI] | [9.74,11.07] | [17.55,22.48] | [13.57,16.27] | <0.001 |
| Stress T4 | [95% CI] | [8.96,10.32] | [19.29,23.17] | [11.97,13.99] | <0.001 |
| baby blues | [95% CI] | [7.29,8.42] | [13.72,17.75] | [11.95,14.21] | <0.001 |

*SM 19: Comparison of the results from the questionnaires of all three subgroups in the “pre-COVID” cohort.*

| **label** | **levels** | **ND**  **(n=157)** | **PPD**  **(n=20)** | **AD**  **(n=34)** | **p value** |
| --- | --- | --- | --- | --- | --- |
| EPDS T0 | [95% CI] | [3.83,4.77] | [8.39,12.8] | [10.42,13.98] | <0.001 |
| EPDS T1 | [95% CI] | [3.88,4.8] | [11.71,17.38] | [8.36,11.34] | <0.001 |
| EPDS T2 | [95% CI] | [2.71,3.58] | [11.11,15.61] | [6.65,9.92] | <0.001 |
| EPDS T3 | [95% CI] | [2.42,3.3] | [10.55,15.18] | [4.97,7.66] | <0.001 |
| EPDS T4 | [95% CI] | [1.87,2.6] | [11.2,15.28] | [4.30,6.45] | <0.001 |
| MPAS T1 | [95% CI] | [85.67,87.32] | [76.65,84.84] | [81.66,86.10] | <0.001 |
| MPAS T2 | [95% CI] | [85.93,87.62] | [73.49,83.52] | [83.27,86.30] | <0.001 |
| MPAS T3 | [95% CI] | [86.7,88.2] | [77.44,85.57] | [84.24,87.63] | <0.001 |
| MPAS T4 | [95% CI] | [87.26,88.77] | [78.08,85.29] | [85.37,88.56] | <0.001 |
| Stress T1 | [95% CI] | [12.35,14.03] | [20.73,26.26] | [16.13,20.45] | <0.001 |
| Stress T2 | [95% CI] | [10.55,12.13] | [18.65,24.96] | [13.93,17.42] | <0.001 |
| Stress T3 | [95% CI] | [9.42,11.07] | [16.47,22.87] | [11.95,15.63] | <0.001 |
| Stress T4 | [95% CI] | [8.56,10.23] | [18.86,23.96] | [11.38,13.85] | <0.001 |
| baby blues | [95% CI] | [7.16,8.53] | [14.02,18.07] | [10.95,14.86] | <0.001 |

*SM 20: Comparison of the results from the questionnaires of all three subgroups in the “lockdown” cohort.*

| **label** | **levels** | **ND**  **(n=75)** | **PPD**  **(n=7)** | **AD**  **(n=34)** | **p value** |
| --- | --- | --- | --- | --- | --- |
| EPDS T0 | [95% CI] | [3.92,5.26] | [4.21,11.49] | [6.75,9.65] | <0.001 |
| EPDS T1 | [95% CI] | [3.55,4.79] | [5.3,16.97] | [8.41,10.87] | <0.001 |
| EPDS T2 | [95% CI] | [2.73,3.88] | [10.09,18.18] | [7.28,9.71] | <0.001 |
| EPDS T3 | [95% CI] | [2.05,3.22] | [8.52,14.90] | [5.71,8.22] | <0.001 |
| EPDS T4 | [95% CI] | [1.75,2.88] | [10.06,15.65] | [4.81,7.47] | <0.001 |
| MPAS T1 | [95% CI] | [83.64,86.49] | [74.46,89.53] | [77.63,83.54] | 0.009 |
| MPAS T2 | [95% CI] | [84.66,87.23] | [62.58,82.84] | [79.46,84.82] | <0.001 |
| MPAS T3 | [95% CI] | [85.51,87.95] | [66.51,92.62] | [82.00,86.05] | 0.005 |
| MPAS T4 | [95% CI] | [86.23,88.35] | [71.81,90.47] | [81.72,86.15] | 0.001 |
| Stress T1 | [95% CI] | [11.63,14.12] | [13.66,30.05] | [17.13,20.27] | <0.001 |
| Stress T2 | [95% CI] | [10.65,12.89] | [15.78,29.92] | [15.69,19.00] | <0.001 |
| Stress T3 | [95% CI] | [9.62,11.89] | [17.04,24.95] | [14.05,18.06] | <0.001 |
| Stress T4 | [95% CI] | [8.97,11.34] | [17.85,23.57] | [11.70,15.00] | <0.001 |
| baby blues | [95% CI] | [6.86,8.87] | [8.2,21.51] | [12.00,14.51] | <0.001 |

*SM 21: Comparison of the questionnaire results of ND and AD in the full cohort.*

|  |  | **Diagnosis** |  |  |
| --- | --- | --- | --- | --- |
| **label** |  | **ND**  **(n=232)** | **AD**  **(n=68)** | **p value** |
| EPDS T0 | [95% CI] | [4.01,4.78] | [8.98,11.42] | <0.001 |
| EPDS T1 | [95% CI] | [3.92,4.65] | [8.8,10.69] | <0.001 |
| EPDS T2 | [95% CI] | [2.85,3.54] | [7.4,9.38] | <0.001 |
| EPDS T3 | [95% CI] | [2.44,3.14] | [5.74,7.54] | <0.001 |
| EPDS T4 | [95% CI] | [1.95,2.57] | [4.92,6.6] | <0.001 |
| MPAS T1 | [95% CI] | [85.31,86.75] | [80.39,84.07] | <0.001 |
| MPAS T2 | [95% CI] | [85.81,87.21] | [81.93,85.00] | <0.001 |
| MPAS T3 | [95% CI] | [86.58,87.85] | [83.67,86.29] | 0.001 |
| MPAS T4 | [95% CI] | [87.17,88.39] | [84.07,86.83] | 0.001 |
| Stress T1 | [95% CI] | [12.4,13.78] | [17.19,19.8] | <0.001 |
| Stress T2 | [95% CI] | [10.84,12.12] | [15.32,17.7] | <0.001 |
| Stress T3 | [95% CI] | [9.74,11.07] | [13.57,16.27] | <0.001 |
| Stress T4 | [95% CI] | [8.96,10.32] | [11.97,13.99] | <0.001 |
| baby blues | [95% CI] | [7.29,8.42] | [11.95,14.21] | <0.001 |

*SM 22: Comparison of the questionnaire results of ND and AD in the “pre-COVID” cohort.*

|  |  | **Diagnosis** |  |  |
| --- | --- | --- | --- | --- |
| **label** |  | **ND**  **(n=157)** | **AD**  **(n=34)** | **p value** |
| EPDS T0 | [95% CI] | [3.83,4.77] | [10.42,13.98] | <0.001 |
| EPDS T1 | [95% CI] | [3.88,4.8] | [8.36,11.34] | <0.001 |
| EPDS T2 | [95% CI] | [2.71,3.58] | [6.65,9.92] | <0.001 |
| EPDS T3 | [95% CI] | [2.42,3.3] | [4.97,7.66] | <0.001 |
| EPDS T4 | [95% CI] | [1.87,2.6] | [4.30,6.45] | <0.001 |
| MPAS T1 | [95% CI] | [85.67,87.32] | [81.66,86.10] | 0.012 |
| MPAS T2 | [95% CI] | [85.93,87.62] | [83.27,86.30] | 0.044 |
| MPAS T3 | [95% CI] | [86.7,88.2] | [84.24,87.63] | 0.093 |
| MPAS T4 | [95% CI] | [87.26,88.77] | [85.37,88.56] | 0.239 |
| Stress T1 | [95% CI] | [12.35,14.03] | [16.13,20.45] | <0.001 |
| Stress T2 | [95% CI] | [10.55,12.13] | [13.93,17.42] | <0.001 |
| Stress T3 | [95% CI] | [9.42,11.07] | [11.95,15.63] | <0.001 |
| Stress T4 | [95% CI] | [8.56,10.23] | [11.38,13.85] | 0.001 |
| baby blues | [95% CI] | [7.16,8.53] | [10.95,14.86] | <0.001 |

*SM 23: Comparison of the questionnaire results of ND and AD in the “lockdown” cohort.*

|  |  | **Diagnosis** |  |  |
| --- | --- | --- | --- | --- |
| **label** |  | **ND**  **(n=75)** | **AD**  **(n=34)** | **p value** |
| EPDS T0 | [95% CI] | [3.92,5.26] | [6.75,9.65] | <0.001 |
| EPDS T1 | [95% CI] | [3.55,4.79] | [8.41,10.87] | <0.001 |
| EPDS T2 | [95% CI] | [2.73,3.88] | [7.28,9.71] | <0.001 |
| EPDS T3 | [95% CI] | [2.05,3.22] | [5.71,8.22] | <0.001 |
| EPDS T4 | [95% CI] | [1.75,2.88] | [4.81,7.47] | <0.001 |
| MPAS T1 | [95% CI] | [83.64,86.49] | [77.63,83.54] | 0.002 |
| MPAS T2 | [95% CI] | [84.66,87.23] | [79.46,84.82] | 0.004 |
| MPAS T3 | [95% CI] | [85.51,87.95] | [82.00,86.05] | 0.019 |
| MPAS T4 | [95% CI] | [86.23,88.35] | [81.72,86.15] | 0.002 |
| Stress T1 | [95% CI] | [11.63,14.12] | [17.13,20.27] | <0.001 |
| Stress T2 | [95% CI] | [10.65,12.89] | [15.69,19.00] | <0.001 |
| Stress T3 | [95% CI] | [9.62,11.89] | [14.05,18.06] | <0.001 |
| Stress T4 | [95% CI] | [8.97,11.34] | [11.70,15.00] | 0.003 |
| baby blues | [95% CI] | [6.86,8.87] | [12.00,14.51] | <0.001 |

*SM 24: Comparison of the questionnaire results of ND and PPD in the full cohort.*

|  |  | **Diagnosis** |  |  |
| --- | --- | --- | --- | --- |
| **label** |  | **ND**  **(n=232)** | **PPD**  **(n=27)** | **p value** |
| EPDS T0 | [95% CI] | [4.01,4.78] | [8.06,11.71] | <0.001 |
| EPDS T1 | [95% CI] | [3.92,4.65] | [11.21,16.11] | <0.001 |
| EPDS T2 | [95% CI] | [2.85,3.54] | [11.73,15.39] | <0.001 |
| EPDS T3 | [95% CI] | [2.44,3.14] | [10.76,14.37] | <0.001 |
| EPDS T4 | [95% CI] | [1.95,2.57] | [11.56,14.72] | <0.001 |
| MPAS T1 | [95% CI] | [85.31,86.75] | [77.72,84.42] | <0.001 |
| MPAS T2 | [95% CI] | [85.81,87.21] | [72.70,81.31] | <0.001 |
| MPAS T3 | [95% CI] | [86.58,87.85] | [76.90,84.36] | <0.001 |
| MPAS T4 | [95% CI] | [87.17,88.39] | [78.3,84.78] | <0.001 |
| Stress T1 | [95% CI] | [12.4,13.78] | [20.44,25.7] | <0.001 |
| Stress T2 | [95% CI] | [10.84,12.12] | [19.37,24.79] | <0.001 |
| Stress T3 | [95% CI] | [9.74,11.07] | [17.55,22.48] | <0.001 |
| Stress T4 | [95% CI] | [8.96,10.32] | [19.29,23.17] | <0.001 |
| baby blues | [95% CI] | [7.29,8.42] | [13.72,17.75] | <0.001 |

*SM 25: Comparison of the questionnaire results of ND and PPD in the “pre-COVID” cohort.*

|  |  | **Diagnosis** |  |  |
| --- | --- | --- | --- | --- |
| **Pre-COVID** |  | **ND**  **(n=157)** | **PPD**  **(n=20)** | **p value** |
| EPDS T0 | [95% CI] | [3.83,4.77] | [8.39,12.8] | <0.001 |
| EPDS T1 | [95% CI] | [3.88,4.8] | [11.71,17.38] | <0.001 |
| EPDS T2 | [95% CI] | [2.71,3.58] | [11.11,15.61] | <0.001 |
| EPDS T3 | [95% CI] | [2.42,3.3] | [10.55,15.18] | <0.001 |
| EPDS T4 | [95% CI] | [1.87,2.6] | [11.2,15.28] | <0.001 |
| MPAS T1 | [95% CI] | [85.67,87.32] | [76.65,84.84] | <0.001 |
| MPAS T2 | [95% CI] | [85.93,87.62] | [73.49,83.52] | <0.001 |
| MPAS T3 | [95% CI] | [86.7,88.2] | [77.44,85.57] | <0.001 |
| MPAS T4 | [95% CI] | [87.26,88.77] | [78.08,85.29] | <0.001 |
| Stress T1 | [95% CI] | [12.35,14.03] | [20.73,26.26] | <0.001 |
| Stress T2 | [95% CI] | [10.55,12.13] | [18.65,24.96] | <0.001 |
| Stress T3 | [95% CI] | [9.42,11.07] | [16.47,22.87] | <0.001 |
| Stress T4 | [95% CI] | [8.56,10.23] | [18.86,23.96] | <0.001 |
| baby blues | [95% CI] | [7.16,8.53] | [14.02,18.07] | <0.001 |

*SM 26: Comparison of the questionnaire results of ND and PPD in the “lockdown” cohort.*

|  |  | **Diagnosis** |  |  |
| --- | --- | --- | --- | --- |
| **lockdown** |  | **ND**  **(n=75)** | **PPD**  **(n=7)** | **p value** |
| EPDS T0 | [95% CI] | [3.92,5.26] | [4.21,11.49] | 0.007 |
| EPDS T1 | [95% CI] | [3.55,4.79] | [5.3,16.97] | <0.001 |
| EPDS T2 | [95% CI] | [2.73,3.88] | [10.09,18.18] | <0.001 |
| EPDS T3 | [95% CI] | [2.05,3.22] | [8.52,14.90] | <0.001 |
| EPDS T4 | [95% CI] | [1.75,2.88] | [10.06,15.65] | <0.001 |
| MPAS T1 | [95% CI] | [83.64,86.49] | [74.46,89.53] | 0.226 |
| MPAS T2 | [95% CI] | [84.66,87.23] | [62.58,82.84] | <0.001 |
| MPAS T3 | [95% CI] | [85.51,87.95] | [66.51,92.62] | 0.006 |
| MPAS T4 | [95% CI] | [86.23,88.35] | [71.81,90.47] | 0.004 |
| Stress T1 | [95% CI] | [11.63,14.12] | [13.66,30.05] | <0.001 |
| Stress T2 | [95% CI] | [10.65,12.89] | [15.78,29.92] | <0.001 |
| Stress T3 | [95% CI] | [9.62,11.89] | [17.04,24.95] | <0.001 |
| Stress T4 | [95% CI] | [8.97,11.34] | [17.85,23.57] | <0.001 |
| baby blues | [95% CI] | [6.86,8.87] | [8.2,21.51] | <0.001 |

*SM 27: Comparison of the questionnaire results of AD and PPD in the full cohort.*

|  |  | **Diagnosis** |  |  |
| --- | --- | --- | --- | --- |
| **label** |  | **PPD**  **(n=27)** | **AD**  **(n=68)** | **p value** |
| EPDS T0 | [95% CI] | [8.06,11.71] | [8.98,11.42] | 0.778 |
| EPDS T1 | [95% CI] | [11.21,16.11] | [8.8,10.69] | <0.001 |
| EPDS T2 | [95% CI] | [11.73,15.39] | [7.4,9.38] | <0.001 |
| EPDS T3 | [95% CI] | [10.76,14.37] | [5.74,7.54] | <0.001 |
| EPDS T4 | [95% CI] | [11.56,14.72] | [4.92,6.6] | <0.001 |
| MPAS T1 | [95% CI] | [77.72,84.42] | [80.39,84.07] | 0.518 |
| MPAS T2 | [95% CI] | [72.70,81.31] | [81.93,85.00] | <0.001 |
| MPAS T3 | [95% CI] | [76.90,84.36] | [83.67,86.29] | 0.006 |
| MPAS T4 | [95% CI] | [78.3,84.78] | [84.07,86.83] | 0.01 |
| Stress T1 | [95% CI] | [20.44,25.7] | [17.19,19.8] | 0.001 |
| Stress T2 | [95% CI] | [19.37,24.79] | [15.32,17.7] | <0.001 |
| Stress T3 | [95% CI] | [17.55,22.48] | [13.57,16.27] | <0.001 |
| Stress T4 | [95% CI] | [19.29,23.17] | [11.97,13.99] | <0.001 |
| baby blues | [95% CI] | [13.72,17.75] | [11.95,14.21] | 0.017 |

*SM 28: Comparison of the questionnaire results of AD and PPD in the” pre-COVID” cohort.*

|  |  | **Diagnosis** |  |  |
| --- | --- | --- | --- | --- |
| **Pre-COVID** |  | **PPD**  **(n=20)** | **AD**  **(n=34)** | **p value** |
| EPDS T0 | [95% CI] | [8.39,12.8] | [10.42,13.98] | 0.256 |
| EPDS T1 | [95% CI] | [11.71,17.38] | [8.36,11.34] | 0.002 |
| EPDS T2 | [95% CI] | [11.11,15.61] | [6.65,9.92] | <0.001 |
| EPDS T3 | [95% CI] | [10.55,15.18] | [4.97,7.66] | <0.001 |
| EPDS T4 | [95% CI] | [11.2,15.28] | [4.30,6.45] | <0.001 |
| MPAS T1 | [95% CI] | [76.65,84.84] | [81.66,86.10] | 0.135 |
| MPAS T2 | [95% CI] | [73.49,83.52] | [83.27,86.30] | 0.004 |
| MPAS T3 | [95% CI] | [77.44,85.57] | [84.24,87.63] | 0.006 |
| MPAS T4 | [95% CI] | [78.08,85.29] | [85.37,88.56] | 0.003 |
| Stress T1 | [95% CI] | [20.73,26.26] | [16.13,20.45] | 0.004 |
| Stress T2 | [95% CI] | [18.65,24.96] | [13.93,17.42] | <0.001 |
| Stress T3 | [95% CI] | [16.47,22.87] | [11.95,15.63] | 0.001 |
| Stress T4 | [95% CI] | [18.86,23.96] | [11.38,13.85] | <0.001 |
| baby blues | [95% CI] | [14.02,18.07] | [10.95,14.86] | 0.036 |

*SM 29: Comparison of the questionnaire results of AD and PPD in the “lockdown” cohort.*

|  |  | **Diagnosis** |  |  |
| --- | --- | --- | --- | --- |
| **lockdown** |  | **PPD**  **(n=7)** | **AD**  **(n=34)** | **p value** |
| EPDS T0 | [95% CI] | [4.21,11.49] | [6.75,9.65] | 0.84 |
| EPDS T1 | [95% CI] | [5.3,16.97] | [8.41,10.87] | 0.382 |
| EPDS T2 | [95% CI] | [10.09,18.18] | [7.28,9.71] | 0.001 |
| EPDS T3 | [95% CI] | [8.52,14.90] | [5.71,8.22] | 0.003 |
| EPDS T4 | [95% CI] | [10.06,15.65] | [4.81,7.47] | <0.001 |
| MPAS T1 | [95% CI] | [74.46,89.53] | [77.63,83.54] | 0.688 |
| MPAS T2 | [95% CI] | [62.58,82.84] | [79.46,84.82] | 0.009 |
| MPAS T3 | [95% CI] | [66.51,92.62] | [82.00,86.05] | 0.171 |
| MPAS T4 | [95% CI] | [71.81,90.47] | [81.72,86.15] | 0.345 |
| Stress T1 | [95% CI] | [13.66,30.05] | [17.13,20.27] | 0.168 |
| Stress T2 | [95% CI] | [15.78,29.92] | [15.69,19.00] | 0.017 |
| Stress T3 | [95% CI] | [17.04,24.95] | [14.05,18.06] | 0.038 |
| Stress T4 | [95% CI] | [17.85,23.57] | [11.70,15.00] | <0.001 |
| baby blues | [95% CI] | [8.2,21.51] | [12.00,14.51] | 0.382 |

*SM 30: Comparison of the questionnaire results of AD women in the “pre-COVID” and “lockdown” cohorts.*

| **AD** |  | **Lockdown**  **(n=34)** | **pre-COVID**  **(n=34)** | **p value** |
| --- | --- | --- | --- | --- |
| EPDS T0 | [95% CI] | [6.75,9.65] | [10.42,13.98] | 0.001 |
| EPDS T1 | [95% CI] | [8.41,10.87] | [8.36,11.34] | 0.829 |
| EPDS T2 | [95% CI] | [7.28,9.71] | [6.65,9.92] | 0.838 |
| EPDS T3 | [95% CI] | [5.71,8.22] | [4.97,7.66] | 0.477 |
| EPDS T4 | [95% CI] | [4.81,7.47] | [4.30,6.45] | 0.366 |
| MPAS T1 | [95% CI] | [77.63,83.54] | [81.66,86.10] | 0.074 |
| MPAS T2 | [95% CI] | [79.46,84.82] | [83.27,86.30] | 0.085 |
| MPAS T3 | [95% CI] | [82.00,86.05] | [84.24,87.63] | 0.146 |
| MPAS T4 | [95% CI] | [81.72,86.15] | [85.37,88.56] | 0.027 |
| Stress T1 | [95% CI] | [17.13,20.27] | [16.13,20.45] | 0.755 |
| Stress T2 | [95% CI] | [15.69,19.00] | [13.93,17.42] | 0.161 |
| Stress T3 | [95% CI] | [14.05,18.06] | [11.95,15.63] | 0.095 |
| Stress T4 | [95% CI] | [11.70,15.00] | [11.38,13.85] | 0.47 |
| baby blues | [95% CI] | [12.00,14.51] | [10.95,14.86] | 0.76 |

*SM 31: Comparison of the questionnaire results of ND women in the “pre-COVID” and “lockdown” cohorts.*

| **ND** |  | **Lockdown**  **(n=75)** | **pre-COVID**  **(n=157)** | **p value** |
| --- | --- | --- | --- | --- |
| EPDS T0 | [95% CI] | [3.92,5.26] | [3.83,4.77] | 0.49 |
| EPDS T1 | [95% CI] | [3.55,4.79] | [3.88,4.8] | 0.67 |
| EPDS T2 | [95% CI] | [2.73,3.88] | [2.71,3.58] | 0.682 |
| EPDS T3 | [95% CI] | [2.05,3.22] | [2.42,3.3] | 0.559 |
| EPDS T4 | [95% CI] | [1.75,2.88] | [1.87,2.6] | 0.802 |
| MPAS T1 | [95% CI] | [83.64,86.49] | [85.67,87.32] | 0.068 |
| MPAS T2 | [95% CI] | [84.66,87.23] | [85.93,87.62] | 0.272 |
| MPAS T3 | [95% CI] | [85.51,87.95] | [86.7,88.2] | 0.292 |
| MPAS T4 | [95% CI] | [86.23,88.35] | [87.26,88.77] | 0.267 |
| Stress T1 | [95% CI] | [11.63,14.12] | [12.35,14.03] | 0.673 |
| Stress T2 | [95% CI] | [10.65,12.89] | [10.55,12.13] | 0.537 |
| Stress T3 | [95% CI] | [9.62,11.89] | [9.42,11.07] | 0.466 |
| Stress T4 | [95% CI] | [8.97,11.34] | [8.56,10.23] | 0.301 |
| baby blues | [95% CI] | [6.86,8.87] | [7.16,8.53] | 0.983 |

*SM 32: Comparison of the questionnaire results of PPD women in the ”pre-COVID” and “lockdown” cohorts.*

| **PPD** |  | **Lockdown**  **(n=7)** | **pre-COVID**  **(n=20)** | **p value** |
| --- | --- | --- | --- | --- |
| EPDS T0 | [95% CI] | [4.21,11.49] | [8.39,12.8] | 0.18 |
| EPDS T1 | [95% CI] | [5.3,16.97] | [11.71,17.38] | 0.217 |
| EPDS T2 | [95% CI] | [10.09,18.18] | [11.11,15.61] | 0.705 |
| EPDS T3 | [95% CI] | [8.52,14.90] | [10.55,15.18] | 0.576 |
| EPDS T4 | [95% CI] | [10.06,15.65] | [11.2,15.28] | 0.833 |
| MPAS T1 | [95% CI] | [74.46,89.53] | [76.65,84.84] | 0.744 |
| MPAS T2 | [95% CI] | [62.58,82.84] | [73.49,83.52] | 0.229 |
| MPAS T3 | [95% CI] | [66.51,92.62] | [77.44,85.57] | 0.732 |
| MPAS T4 | [95% CI] | [71.81,90.47] | [78.08,85.29] | 0.878 |
| Stress T1 | [95% CI] | [13.66,30.05] | [20.73,26.26] | 0.583 |
| Stress T2 | [95% CI] | [15.78,29.92] | [18.65,24.96] | 0.724 |
| Stress T3 | [95% CI] | [17.04,24.95] | [16.47,22.87] | 0.62 |
| Stress T4 | [95% CI] | [17.85,23.57] | [18.86,23.96] | 0.757 |
| baby blues | [95% CI] | [8.2,21.51] | [14.02,18.07] | 0.603 |

*SM 33: Comparison of the EPDS scores between the diagnoses across the cohorts and diagnoses, significant at the uncorrected p<0.001.*

| EPDS | | T0 | T1 | T2 | T3 | T4 |
| --- | --- | --- | --- | --- | --- | --- |
| ND vs AD | Full,  “pre-COVID” & “lockdown”  cohorts | AD>ND | | | | |
| ND vs PPD | Full &  “pre-COVID”  cohorts | PPD>ND | | | | |
|  | “Lockdown”  cohort | No difference, but a tendency (p=0.007) towards PPD>ND | PPD>ND | | | |
| PPD vs AD | Full cohort | No difference | PPD>AD | | | |
|  | “pre-COVID” cohort | No difference, but a tendency at T1 (p=0.002) towards PPD>AD | | PPD>AD | | |
|  | “Lockdown” cohort | No difference | | PPD>AD | No difference, but a tendency (p=0.003) towards PPD>AD | PPD>AD |

*SM 34: Comparison of the EPDS scores between “pre-COVID” and “lockdown” cohorts within the diagnostic groups.*

| EPDS | T0 | T1 | T2 | T3 | T4 |
| --- | --- | --- | --- | --- | --- |
| ND | No difference | | | | |
| AD | “pre-COVID”  >  “lockdown” | No difference | | | |
| PPD | No difference | | | | |

*SM 35: Comparison of the MPAS scores between the diagnoses across the cohorts and diagnoses.*

| MPAS | | T1 | T2 | T3 | | T4 | |
| --- | --- | --- | --- | --- | --- | --- | --- |
| ND vs AD | Full cohort | AD<ND | | | | | |
|  | “Pre-COVID” cohort | No difference | | | | | |
|  | “Lockdown” cohort | No difference, but a tendency for the “lockdown” cohort at all time points towards AD<ND | | | | | |
| ND vs PPD | Full &  “pre-COVID” cohorts | PPD<ND | | | | | |
|  | “Lockdown” cohort | No difference | PPD<ND | No difference, but a tendency for T3 and T4 with p=0.006 and 0.004 towards PPD<ND | | | |
| PPD vs AD | Full cohort | No difference | PPD<AD | No difference | | | No difference, but a tendency with p=0.006 towards PPD<AD |
|  | “pre-COVID” cohort | No difference | No difference, but a tendency for T2-T4 with p=0.004, 0.006, 0.003 towards PPD<AD | | | | |
|  | “Lockdown” cohort | No difference | No difference, but a tendency with p=0.009 towards PPD<AD | | No difference | | |

*SM36: Comparison of the PSS scores between the diagnoses across the cohorts and diagnoses.*

| PSS | | T1 | T2 | T3 | T4 |
| --- | --- | --- | --- | --- | --- |
| ND vs AD | Full &  “pre-COVID” cohorts | AD>ND | | | |
|  | “Lockdown” cohort | AD>ND | | | No difference,  but tendency (p=0.003) |
| ND vs PPD | Full,  “pre-COVID” &  “lockdown”cohorts | PPD>ND | | | |
| PPD vs AD | Full cohort | PPD>AD | | | |
|  | “pre-COVID” cohort | No difference,  but tendency (p=0.004) | PPD>AD | | |
|  | “Lockdown” cohort | No difference | | | PPD>AD |
